# Ring Contraction of Cyclooctatetraenes toward Non‐Benzenoid Polycyclic Aromatic Hydrocarbons by Au(111)‐Catalysis and Bulk Pyrolysis

**DOI:** 10.1002/chem.202501101

**Published:** 2025-06-27

**Authors:** Svenja Weigold, Ye Liu, Hailong Li, Qiang Chen, Xuechao Li, Haiming Zhang, Miao Xie, Frank Rominger, Jan Freudenberg, Uwe H. F. Bunz, Klaus Müllen, Lifeng Chi

**Affiliations:** ^1^ Max‐Planck‐Institut für Polymerforschung Ackermannweg 10 55128 Mainz Germany; ^2^ Organisch‐Chemisches Institut, Ruprecht‐Karls‐Universität Heidelberg Im Neuenheimer Feld 270 69120 Heidelberg Germany; ^3^ State Key Laboratory of Bioinspired Interfacial Materials Science, Institute of Functional Nano & Soft Materials (FUNSOM) Jiangsu Key Laboratory for Carbon‐Based Functional Materials and Devices Joint International Research Laboratory of Carbon‐Based Functional Materials and Devices, Soochow University Ren'ai Road 199 Suzhou 215123 China

**Keywords:** cyclooctatetraenes, non‐benzenoid polycyclic aromatic hydrocarbons, ring contraction, ring rearrangement, scanning probe microscope

## Abstract

The Au(111)‐catalyzed cyclodehydrogenation of tetraphenylated diacenaphtho‐cyclooctatetraene (**DA‐COT**) and tetraacenaphtho‐cyclooctatetraene (**TA‐COT**) was investigated and compared with their uncatalyzed bulk pyrolysis. On Au(111), a strain‐induced contraction of the central COT unit toward six‐membered rings upon extrusion of alkynes occurred. For **TA‐COT**, an alternative reaction pathway opened up due to the increased ring strain of the eliminated alkyne product. This pathway involved a rearrangement toward a non‐benzenoid polycyclic aromatic hydrocarbon containing a sesquifulvalene core. Intermediates and (by‐)products were observed by high‐resolution scanning tunneling microscopy (STM) and non‐contact atomic force microscopy (nc‐AFM), verifying the predicted reaction mechanism. Formation of [8]circulene derivatives was not observed. Uncatalyzed bulk pyrolysis only gave one ring contraction product, respectively, for both COT derivatives.

## Graphical abstract

The Au(111)‐catalyzed cyclodehydrogenation of tetraphenylated **DA‐COT** and **TA‐COT** is compared with bulk pyrolysis. On Au(111), a strain‐induced contraction of the central COT unit toward six‐membered rings occurs. For **TA‐COT**, a rearrangement toward a non‐benzenoid polycyclic aromatic hydrocarbon containing five‐ and seven‐membered rings is observed as an alternative reaction product. Uncatalyzed bulk pyrolysis only gives the ring contraction product for both COT derivatives.

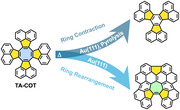

## 1 Introduction

Polycyclic aromatic hydrocarbons (PAHs), which can be regarded as molecularly defined graphene segments,^[^ ^1^ ^]^ have garnered widespread attention due to their great potential for applications in (opto)electronics^[^ ^2^, ^3^, ^4^ ^]^ or spintronics.^[^ ^5^, ^6^, ^7^, ^8^ ^]^ Their electronic properties are influenced by the number and connectivity of hexagonal rings^[^ ^9^, ^10^, ^11^, ^12^ ^]^ as well as by the introduction of non‐benzenoid rings.^[^ ^13^, ^14^, ^15^, ^16^, ^17^, ^18^ ^]^ While the synthesis of benzenoid PAHs has been well studied,^[^ ^1^, ^10^, ^11^, ^12^, ^19^, ^20^, ^21^, ^22^ ^]^ that of PAHs containing non‐benzenoid rings still poses great challenges.^[^ ^16^, ^23^, ^24^, ^25^ ^]^ Incorporating cyclooctatetraene (COT) into PAHs based on negatively curved [8]circulenes, in which the central COT ring is surrounded by eight annulated benzene rings, has proven especially difficult. This is due to the high ring strain of COT‐containing PAHs.^[^ ^25^, ^26^, ^27^, ^28^, ^29^, ^30^, ^31^ ^]^ Bottom‐up synthesis using oxidative cyclodehydrogenation reactions has been established as a strategy for the formation of non‐benzenoid PAHs containing octagonal rings.^[^ ^23^, ^32^, ^33^, ^34^, ^35^ ^]^ This approach, however, has been hampered by rearrangements, including ring contractions toward less strained benzenoid or non‐benzenoid PAHs.^[^ ^28^, ^36^, ^37^, ^38^, ^39^ ^]^

Thermally activated reactions on metal surfaces under ultrahigh vacuum (UHV) have allowed, among others, C‐H activation reactions or Ullmann couplings and thus largely extended the toolbox of benzenoid or non‐benzenoid PAH synthesis.^[^ ^11^, ^13^, ^16^, ^22^, ^24^, ^40^, ^41^, ^42^ ^]^ Thereby, the metal surface served as a catalyst and stabilized highly reactive products that would not persist in solution.^[^ ^11^, ^13^, ^40^, ^41^, ^43^, ^44^ ^]^ The interaction between adsorbed PAHs and the metal surface encourages planarization of reactants and products; however, access to PAHs containing octagonal rings via metal surfaces remains scarce.^[^ ^45^, ^46^, ^47^, ^48^ ^]^ We are aware of only one publication describing the cyclodehydrogenation of a COT‐containing precursor toward a diaza[8]circulene.^[^ ^49^ ^]^ Recently, a ring contraction of a benzannulated COT into a cyclopenta[*c,d*]azulene on Au(111) was reported.^[^ ^50^ ^]^

**Scheme 1:**
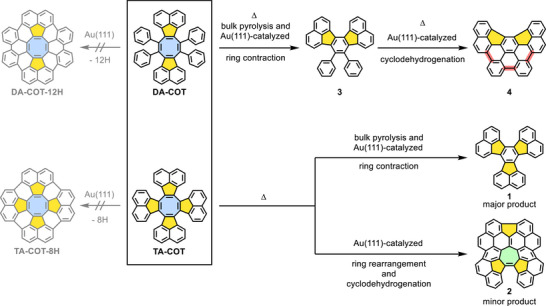
Strain‐induced ring contraction and rearrangement of **DA‐COT** (top) and **TA‐COT** (bottom) via bulk pyrolysis or Au(111)‐catalysis investigated in this work.

Herein, we investigated the cyclodehydrogenation of benzo‐fused COT derivatives on Au(111) under UHV conditions, aiming at the formation of the corresponding [8]circulene derivatives, and compared these reactions with uncatalyzed bulk pyrolysis (see Scheme 1). The starting compounds were two different acenaphtho‐COT derivatives: tetraphenylated diacenaphto‐COT **DA‐COT** and tetraacenaphtho‐annulated COT **TA‐COT**.^[^ ^51^ ^]^ Instead of the formation of the desired [8]circulene derivatives **DA‐COT‐12H** and **TA‐COT‐8H**, strain‐induced rearrangements occurred, resulting in ring contraction of the central COT rings. Tetraphenylated diacenaphtho‐cyclooctatetraene (**DA‐COT**) produced a new six‐membered ring upon extrusion of diphenylacetylene (tolane). Annulation of diacenaphtho‐COT with two additional, sterically demanding acenaphthylene units, as in tetraacenaphtho‐cyclooctatetraene (**TA‐COT**), rendered alkyne elimination less favorable. Thus, the central COT unit transformed into a heptagonal ring with cyclodehydrogenation to the sesquifulvalene derivative **2** due to an alternative reaction pathway on Au(111). Intermediates and (by‐)products were visualized by scanning tunneling microscopy (STM) and non‐contact atomic force microscopy (nc‐AFM), and a reaction mechanism calculated for the thermal rearrangement of cyclooctadi‐*as*‐indacene derivatives^[^ ^52^ ^]^ upon flash vacuum pyrolysis (FVP)^[^ ^53^ ^]^ was suggested. The electronic characteristics of the ring contraction and rearrangement products were also probed using scanning tunneling spectroscopy (STS). Density functional theory (DFT) computations revealed partial electron transfer from the adsorbed sesquifulvalene **2** onto Au(111). Differing from the metal‐catalyzed reactions, bulk pyrolysis of **DA‐COT** and **TA‐COT** only furnished contraction of the central COT ring under alkyne extrusion for both COT derivatives.

## 2 Results and Discussion

### 2.1 Synthesis

**Scheme 2:**
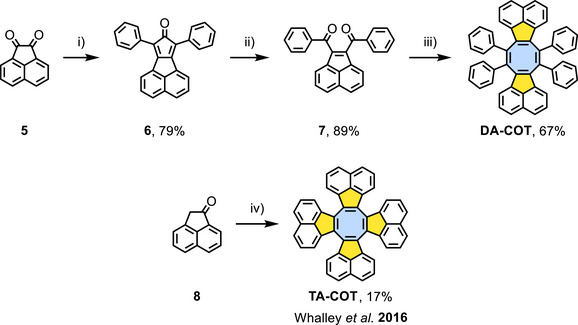
Synthesis of **DA‐COT** and **TA‐COT**. Conditions: i) 1,3‐diphenylacetone, KOH, EtOH, reflux, 15 minutes; ii) H_2_O_2_, Na_2_MoO_4_, SDS, DCM/*n*‐BuOH/H_2_O (10:4:1, *v*/*v*/*v*), rt, 2 days; iii) TiCl_4_, Zn, THF, 0 °C to reflux, overnight; iv) TiCl_4_, *o*‐dichlorobenzene, reflux, 2 hours.^[^ ^51^ ^].^

The synthesis of **DA‐COT** (Scheme 2) started from commercially available acenaphthoquinone (**5**). A twofold Knoevenagel condensation of **5** with 1,3‐diphenylacetone gave the literature‐known cyclopentadienone **6** (79% yield).^[^ ^54^ ^]^ **6** was oxidized with singlet oxygen generated catalytically from hydrogen peroxide in a multiphase system with sodium dodecyl sulfate (SDS) as surfactant^[^ ^55^ ^]^ furnishing 1,4‐diketone **7** (89%). McMurry coupling of **7** generated **DA‐COT** in 67% yield (see SI for experimental procedures and characterization). **TA‐COT** was obtained following an established protocol via Lewis‐acid‐catalyzed Aldol cyclization of acenaphthylen‐1(2*H*)‐one (**8**).^[^ ^51^, ^56^, ^57^ ^]^

### 2.2 Ring Contraction of **DA‐COT** on Surface

**Figure 1:**
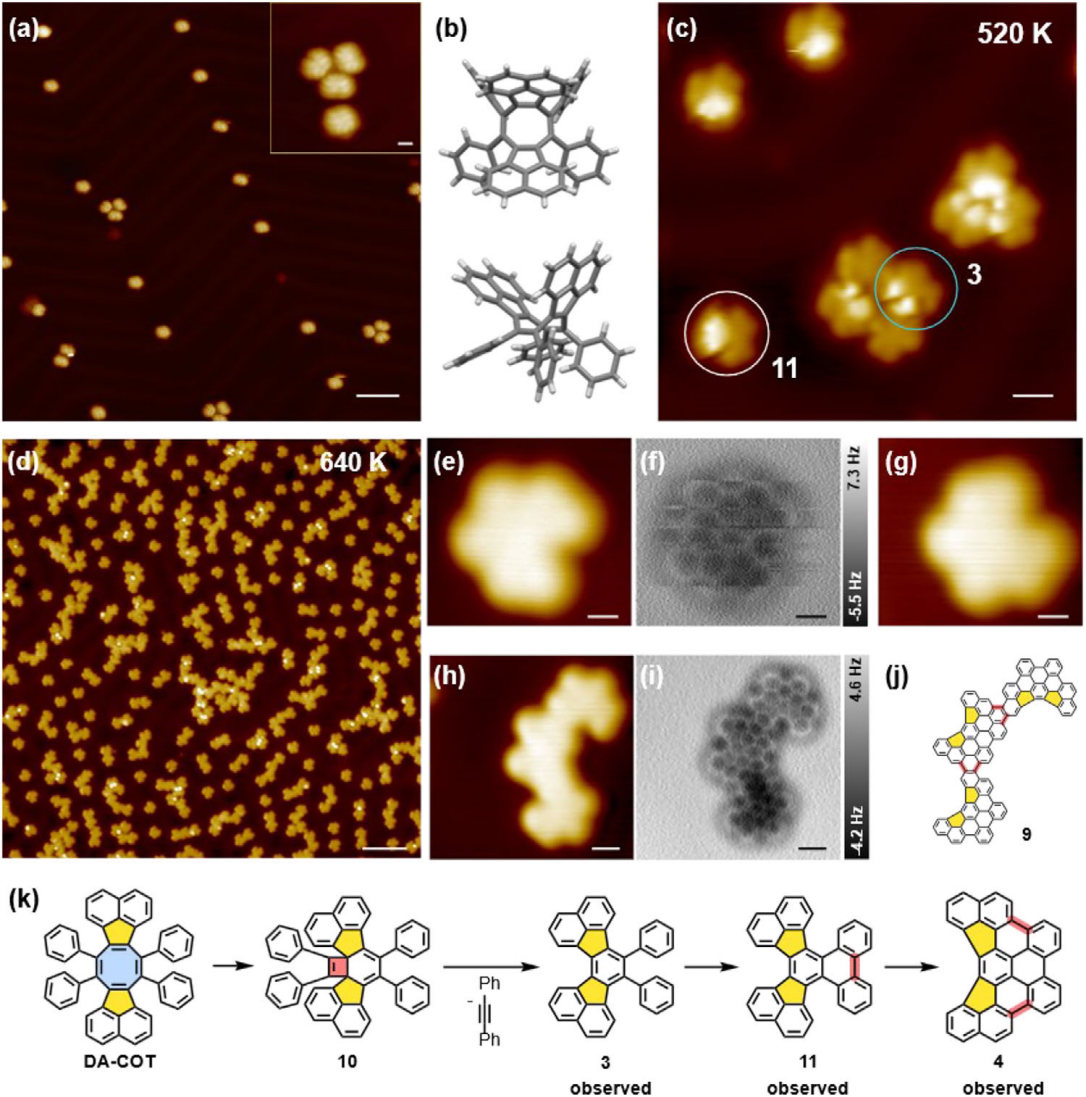
Ring contraction of **DA‐COT** on Au(111). a) Large‐scale STM images of **DA‐COT** at RT; b) top and side views of crystal structure of **DA‐COT** (capped sticks model), CCDC number 2399881; c) STM images after annealing **DA‐COT** to 520 K (blue circle: **3**, white circle: **11**); d) reaction of **DA‐COT** on Au(111) at 640 K; e)–g) STM images and constant‐height AFM image of ring‐contraction product **4** e) before and g) after nc‐AFM measurement); h)–j) STM images, atomically resolved nc‐AFM images, and chemical structure of trimer **9**; k) proposed mechanism for ring contraction and cyclodehydrogenation of **DA‐COT**. STM scanning parameters: a), c) V_bias_ = −2 V, I = 3 pA; d) V_bias_ = −200 mV, I = 5 pA; e), g), h) V_bias_ = −50 mV, I = 8 pA. nc‐AFM imaging heights: f) ∆Z = 0 pm; i) ∆Z = 50 pm. Δz refers to the vertical displacement of the AFM tip relative to the STM setpoint position. Scale bars: a) 5 nm, inset of a) 0.8 nm; c) 1 nm; d) 5 nm; e)‐g) 0.3 nm; h), i) 0.5 nm.

After deposition of precursor **DA‐COT** onto an Au(111) surface from a Knudsen cell at an evaporation temperature of 470 K, the surface coverage was monitored by STM with a CO‐functionalized tip. As shown in Figure 1a, the molecules physisorbed predominantly as unimers together with some clusters of two or three monomeric units (see inset of Figure 1a). The adsorbates were not fully planar, consistent with the tub shape of COT (see crystal structure in Figure 1b). After annealing at 520 K, the central COT unit contracted into a benzene ring, forming the triangular product **3** (blue circle in Figure 1c) as well as **11** (white circle in Figure 1c). Compared to **3**, hydrocarbon **11** is partially cyclodehydrogenated. The STM image of **3** displays two spots with higher density of electronic states attributed to the two phenyl groups. The region of lower density of states represents the planar diacenaphtho‐benzene subunit of **3**. Within product **11** the contrast is stronger, suggesting a non‐planar conformation of **11**. The STM simulations of molecules **3** and **11** shown in Figure S9 are in good agreement with the experimental observations.

After deposition of **DA‐COT** onto the Au(111) surface preheated to 640 K, the ring contracted, and fully cyclodehydrogenated product **4**, together with oligomers, was formed (Figure 1d–g). Due to weak physisorption of **4** on the Au(111) surface as well as short‐range repulsive interactions between the CO‐functionalized tip and the molecule in the constant‐height AFM mode, molecules changed their position and orientation during nc‐AFM measurements (Figure 1e,g). Therefore, the structure of **4** could not be solved clearly using nc‐AFM (Figure 1f). Nevertheless, oligomers of **4**, exhibiting a lower mobility than **4** due to stronger physisorption, appeared as adequate model compounds for analyzing the structure of **4**. By considering trimer **9**, consisting of three monomeric subunits of **4** (Figure 1h–j), the chemical structure of monomer **4** could be clearly identified as a cyclodehydrogenated acenaphthotriphenylene according to the reaction scheme in Figure 1k. Based on the observed intermediates and products, the following mechanism for the formation of **4** was proposed: First, the central COT unit of **DA‐COT** undergoes an electrocyclic reaction^[^ ^58^, ^59^ ^]^ to bicyclo[4.2.0]octatriene **10** (not experimentally observed); subsequent retro‐[2 + 2]cycloreversion results in extrusion of diphenylacetylene (tolane) and furnishes **3** with a central benzene unit. The ring contraction is followed by a stepwise cyclodehydrogenation, in which **11** with a phenanthrene subunit is first formed, finally giving the fully fused product **4** (Figure 1k). During ring contraction, two different alkyne elimination products are possible, namely cycloalkyne acenaphthyne (**S4**) and tolane (see Scheme S1). For **DA‐COT**, ring contraction occurred selectively, while the formation of tetraphenylfluoranthene **S5** under extrusion of the highly strained acenaphthyne (**S4**) was not observed.

### 2.3 Ring Contraction and Rearrangement of **TA‐COT**

**Figure 2:**
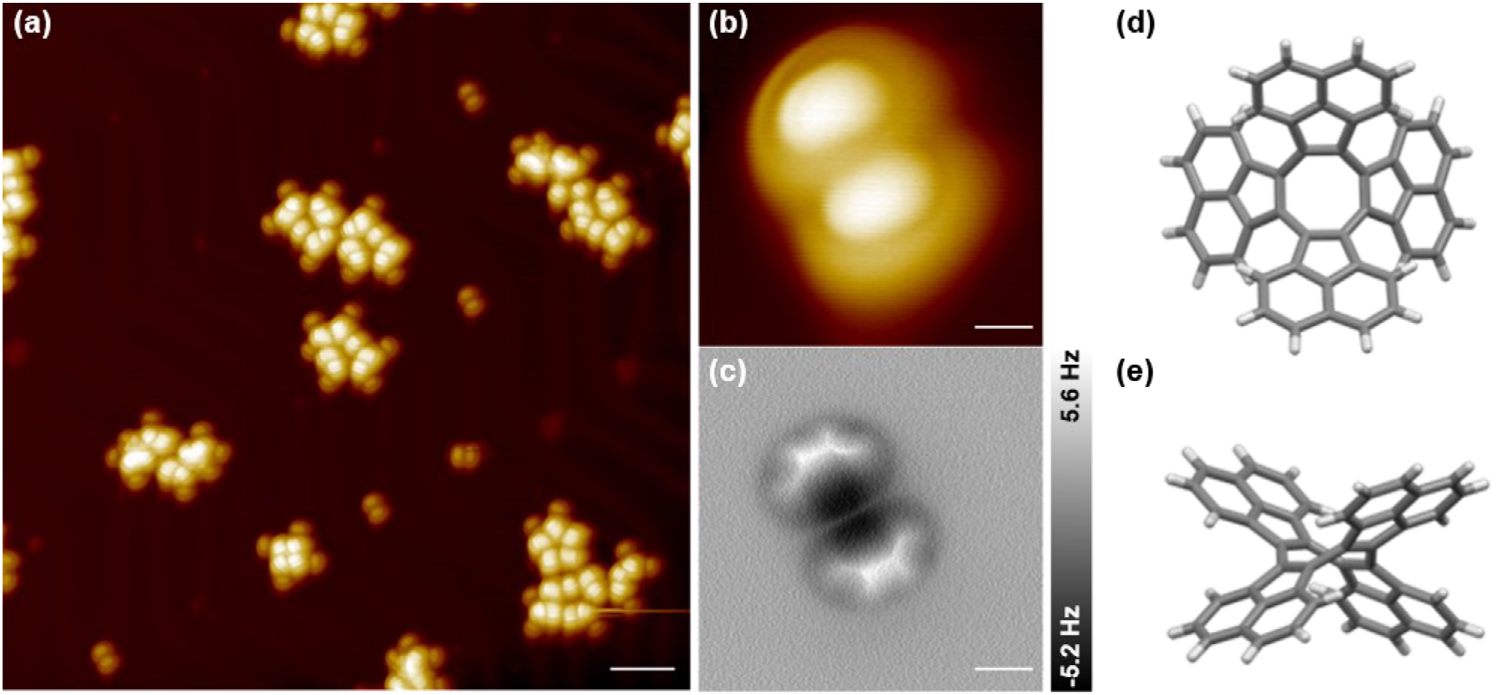
Adsorption of **TA‐COT** on Au(111) at RT. a) Large‐scale STM image of **TA‐COT** on Au(111); b), c) STM image and corresponding nc‐AFM image of an individual **TA‐COT**; d), e) top and side views of crystal structure (capped sticks model, CCDC 1440430).^[^ ^51^ ^]^ STM scanning parameters: a), b) V_bias_ = −2 V, I = 20 pA; nc‐AFM imaging height: c) ∆Z = 0 pm. Scale bars: a) 5 nm; b), c) 0.5 nm.

In order to suppress the undesirable ring contraction of the COT core, the four phenyl substituents of **DA‐COT** were replaced by 1,8‐naphthylene units in **TA‐COT**. Therefore, the formation of strained bicyclo[4.2.0]octatriene **S6** and acenaphthyne (**S4**) (see SI, Figure S8 for strain energy calculations) should be strongly hindered. **TA‐COT** was deposited on Au(111) by organic molecular beam epitaxy, and the adsorption process was monitored via STM (see Figure 2). For the majority of molecules, cluster formation was observed, while a minority adsorbed as unimers on the herringbone reconstructed ridges (Figure 2a). Due to the non‐planar structure of the tub‐shaped COT core (see Figure 2d,e for the crystal structure of **TA‐COT**)^[^ ^51^ ^]^ STM images revealed a pronounced contrast in molecular electronic states (Figure 2b), which was also observed in the corresponding high‐resolution nc‐AFM images of **TA‐COT** (Figure 2c). Not surprisingly, the acenaphtylene units facing the surfaces as well as the COT ring could not be resolved, and the micrographs only provided an incomplete molecular structure.

**Figure 3:**
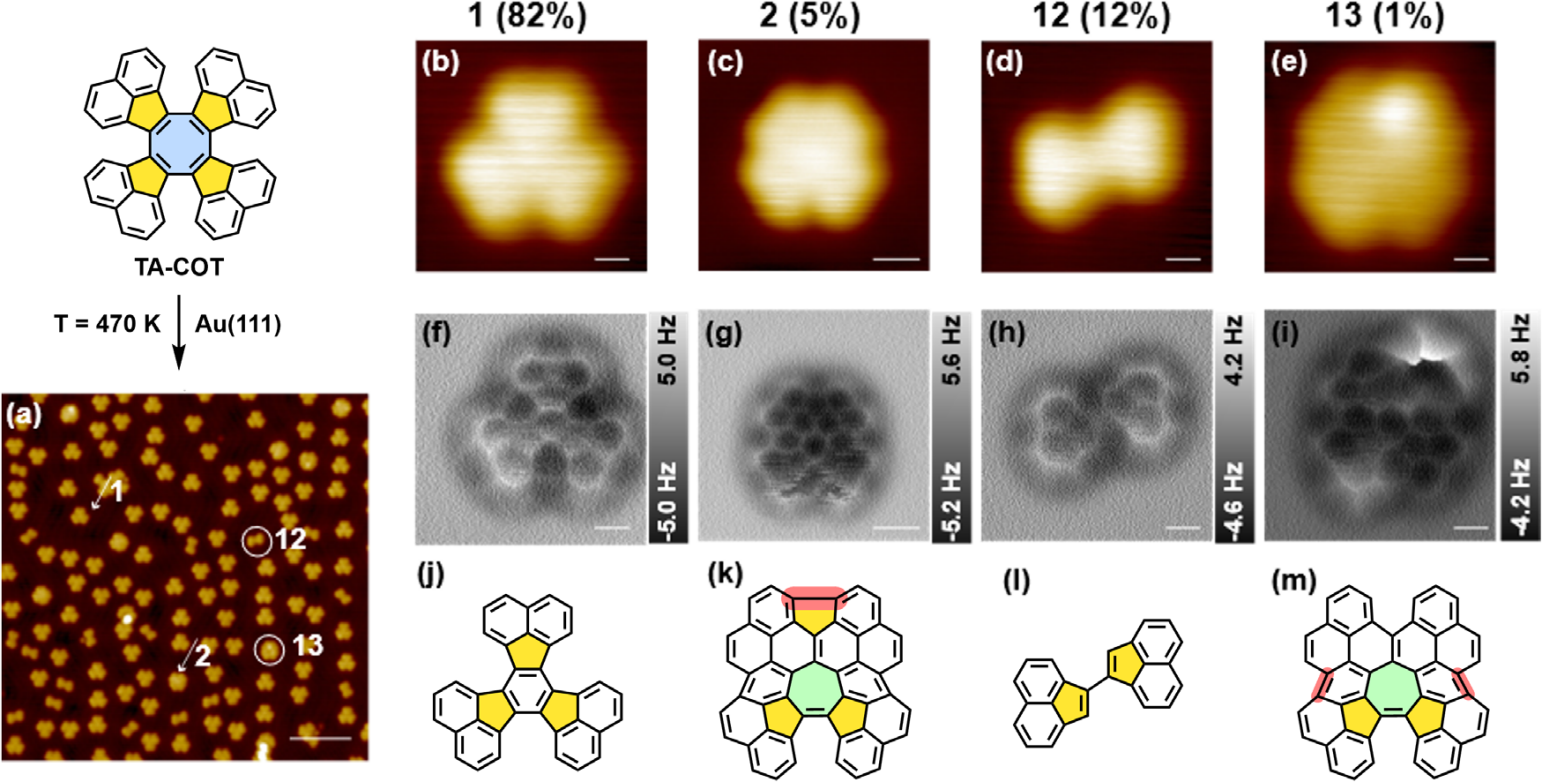
Ring contraction and rearrangement reactions of **TA‐COT** on Au(111) after annealing to 470 K. a) Large‐scale STM images of products **1**, **2**, **12**, and **13** (indicated by white arrows or circles); b)–e) high‐resolution STM images of a) ring contraction product **1**; c) ring rearrangement product **2**; d) dimerization products **12** of extruded substituents after ring contraction; e) heptafulvene **13**. f)–m) Corresponding high‐resolution constant height nc‐AFM images (f–i) and molecular structures (j–m) of **1**, **2**, **12**, and **13**. STM scanning parameters: a), b), d), e) V_bias_ = −200 mV, I = 5 pA; c) V_bias_ = −50 mV, I = 10 pA. nc‐AFM imaging heights: f) ∆Z = −20 pm, g) ∆Z = 20 pm, h) ∆Z = −10 pm, and i) ∆Z = 0 pm. Scale bars: a) 5 nm; b), f), d), h), e), i) 0.3 nm; c), g) 0.5 nm.

After annealing **TA‐COT** at 470 K, several products, namely **1**, **2**, **12**, and **13** (ratio **1**:**2**:**12**:**13** = 82:5:12:1), were visualized by high‐resolution STM and nc‐AFM (see Figure 3). The main product was the D_3h_‐symmetric diacenaphthofluoranthene **1** (Figure 3b,f,j), resulting from a ring contraction of the central COT into a benzene unit. This is analogous to the previously discussed ring contraction of **DA‐COT**. Detection of acenaphthylenyl dimer **12** (Figure 3d,h,l) supported the proposed mechanism (see SI, Scheme S1) via electrocyclic reaction and subsequent cycloalkyne (acenaphthyne **S4**) elimination. Furthermore, a rearrangement of the central 5–8–5 unit with accompanying cyclodehydrogenation (indicated by red bonds in Figure 3k) to sesquifulvalene derivative **2** (Figure 3c,g,k) was observed. This alternative reaction pathway is a consequence of the thermodynamically highly unfavorable ring contraction from **TA‐COT** to **1** and **S4** necessitating at least (neglecting activation barriers) 383 kJ•mol^−1^. The structure of **13** could be assigned to a partly cyclodehydrogenated rearrangement product containing a heptafulvene unit (Figure 3e,i). STM simulations as well as AFM simulations at different tip heights (Figure S10) are in good agreement with the experimental data, confirming the molecular structure of **13**. A mechanism for the sesquifulvalene formation is proposed in Scheme S2. It involves an isomerization of **TA‐COT** into COT **S7** and then rearrangement to carbene **S8**. A twofold cyclodehydrogenation of **S8** furnishes carbene **S9** (formed bonds are marked in red). Subsequently, **S9** rearranges into the observed heptafulvene **13** under expansion of a five‐ into a six‐membered ring. The last step represents a further cyclodehydrogenation under the formation of a pentagonal ring, yielding sesquifulvalene **2**. An equivalent cationic mechanism was postulated in the rearrangement of corresponding COT derivatives under Scholl conditions by Tobe et al.^[^ ^28^ ^]^ Furthermore, an analogous mechanism involving a carbene intermediate was calculated for the thermal rearrangement of cyclooctadi‐*as*‐indacene derivatives under FVP conditions.^[^ ^52^ ^]^

Prompted by the different reactivities of gold and silver surfaces, cyclodehydrogenation of **TA‐COT** was also investigated on Ag(111) and Ag(100) (see SI, Figures S5 and S6). On Ag(111), the formation of ring contraction product **1** as the main product as well as sesquifulvalene **2** was observed after annealing to 470 K. Furthermore, a single cyclodehydrogenation reaction of **TA‐COT** furnished COT derivative **S1** as a minor product. The coverage‐dependent product distribution after annealing to 470 K was investigated and also studied on an Ag(110) surface (see SI, Figure S6). At low coverage, only ring contraction product **1** was formed (Figure S6a), while high coverage (Figure S6b) yielded **1** as the main product and sesquifulvalene **2** as a by‐product. Singly and doubly cyclodehydrogenated products **S1** and **S2** were observed by STM and nc‐AFM.

Thus, when adsorbing **TA‐COT** on either gold or silver surfaces, a ring contraction of the COT unit could not be avoided, and formation of the [8]circulene derivative octadehydrogenated **TA‐COT** (**TA‐COT‐8H**) was not observed. Nevertheless, the rearrangement of **TA‐COT** to sesquifulvalene **2** verified a successful increase of the reaction barriers toward bicyclo[4.2.0]octatriene intermediate and alkyne elimination product, attributable to an increase in strain energies.

### 2.4 Electronic Characterization of Non‐Benzenoid PAHs **4**, **1**, and **2**

**Figure 4:**
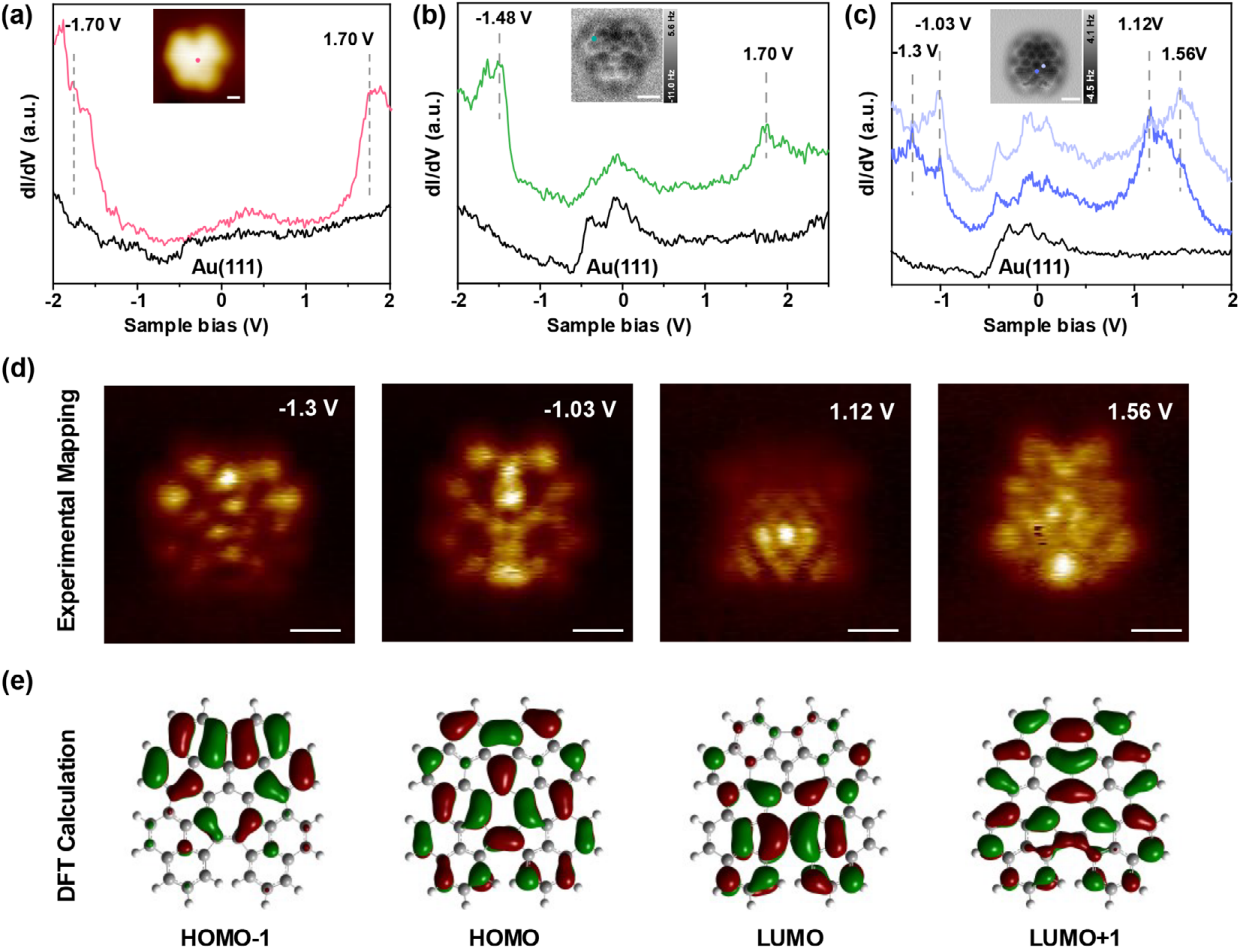
Electronic characterization of products **4**, **1**, and **2** by STS. a) d*I*/d*V* spectra of **4** (red) and Au(111) (black). Inset: high‐resolution STM image of **4**; the STS measurement position is marked in red. b) d*I*/d*V* spectra of 1 (green) and Au(111) (black). Inset: atomically resolved nc‐AFM image of **1**; the STS measurement position is marked in green. c) d*I*/d*V* spectra of **2** (blue) and Au(111) (black). Inset: atomically resolved nc‐AFM image of **2**; the STS measurement positions are marked in blue. d) Constant height d*I*/d*V* mappings of **2** at four different energies. e) Molecular orbital distribution of HOMO‐1, HOMO, LUMO, and LUMO+1 of **2** determined by DFT calculations. STM scanning parameters: a) V_bias_ = −200 mV, I = 5 pA. nc‐AFM imaging heights: b) ∆Z = −30 pm; c) ∆Z = 20 pm. Scale bars: a) 0.3 nm; b)–d) 0.5 nm.

To understand the electronic properties of reaction products **4**, **1**, and **2** and to investigate the influence of seven‐ and five‐membered rings on the electronic states of non‐benzenoid PAHs, STS measurements were conducted (see Figure 4). The differential spectra d*I*/d*V* of **4** (red line), **1** (green line), **2** (blue lines), and bare Au(111) (black line) for comparison are presented in Figure 4a‐c. For cyclodehydrogenated acenaphthotriphenylene **4**, two prominent resonance peaks were observed in the spectra at −1.70 V and 1.70 V (Figure 4a), which could be assigned to the Highest Occupied Molecular Orbital (HOMO) and the Lowest Unoccupied Molecular Orbital (LUMO), respectively. For triacenaphtylenene **1**, resonance peaks at −1.48 V (HOMO) and 1.70 V (LUMO) were obtained (Figure 4b), thus exhibiting a narrower HOMO‐LUMO gap than **4**. In contrast, for sesquifulvalene derivative **2**, four significant resonance peaks were observed in the d*I*/d*V* spectra, centered at −1.30 V, −1.03 V, 1.12 V, and 1.56 V (Figure 4c), respectively. To assign these energy values to electronic states, constant height d*I*/d*V* mappings were measured at the corresponding values (Figure 4d) and compared to DFT calculations of the free‐standing molecule (Figure 4e). The peak at an energy value of −1.30 V could be assigned to HOMO‐1, which was located in the upper half of **2** at the sesquifulvalene unit and their fused benzenoid rings. At −1.03 V (HOMO), the electron density is delocalized over the entire molecule with the highest values at the sesquifulvalene unit. The resonance peaks at energies of 1.12 V and 1.56 V were ascribed to LUMO and LUMO+1, respectively. The corresponding electron density maps reveal that the LUMO is located at the 5–7–5 unit in the lower part of the molecule, while the LUMO+1 is fully delocalized. In total, HOMO‐LUMO gaps of the discussed compounds were calculated as 3.40 eV (**4**), 3.18 eV (**1**), and 2.15 eV (**2**) from experimentally determined values. Introduction of non‐benzenoid five‐ and seven‐membered rings into PAHs thus decreases HOMO‐LUMO gaps. STS spectroscopy of single molecules accordingly confirms the results of classical UV/vis spectroscopy.^[^ ^60^ ^]^

### 2.5 Aromaticity of Rearranged Compound **2** and Its Charge Transfer Interaction With Au(111)

**Figure 5:**
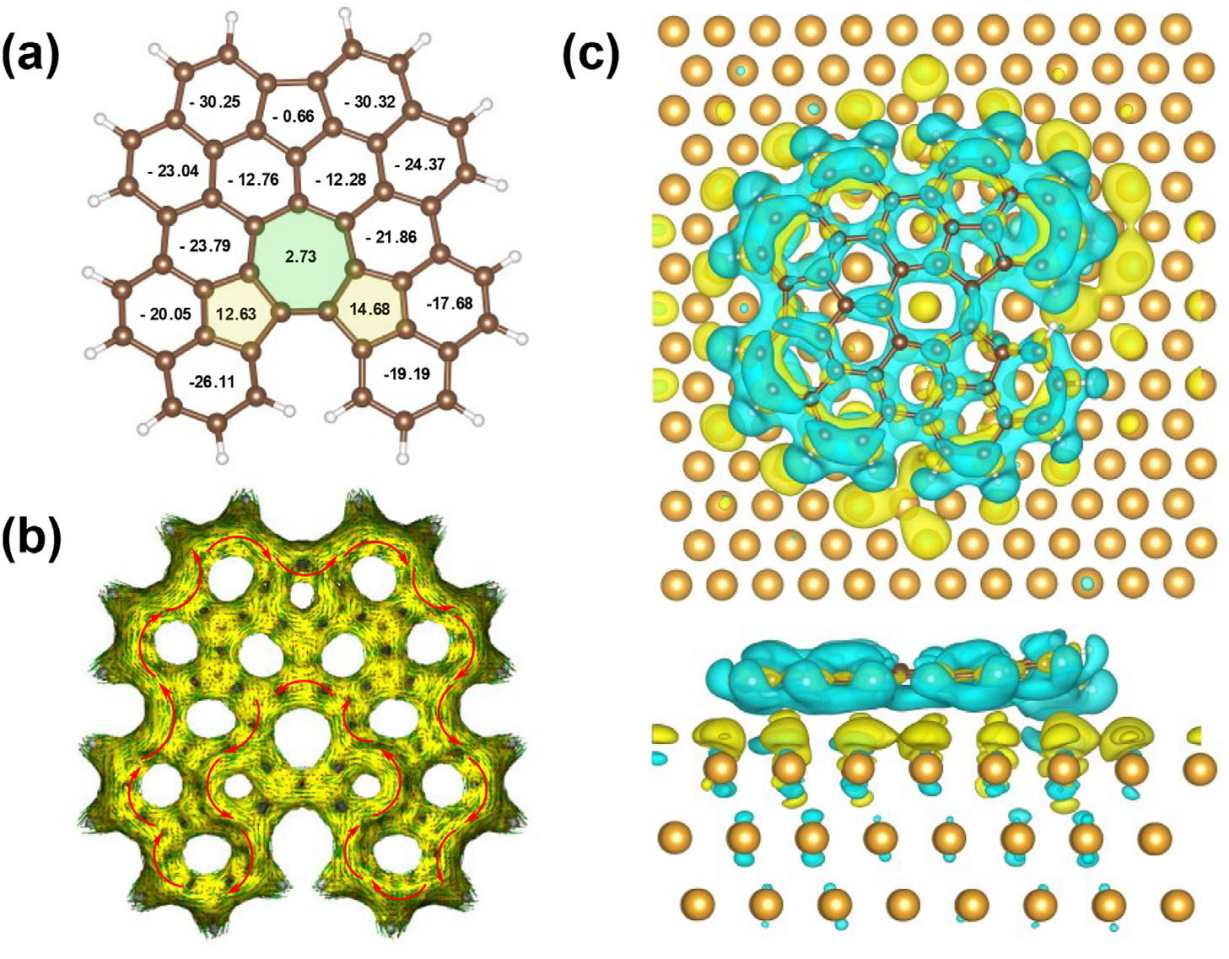
Analysis of the aromaticity of product **2** and charge transfer between **2** and the Au(111) surface. a) NICS(1)_zz values for **2**. b) ACID plot of **2**; the red arrows indicate the ring current flow magnetic field, and the magnetic field vector is oriented out of plane. c) Differential charge density analysis of **2** (top and side view); cyan and yellow colors indicate charge dissipation and accumulation, respectively.

To investigate the aromaticity of the non‐benzenoid PAH **2**, nucleus‐independent chemical shift (NICS) values^[^ ^61^, ^62^, ^63^ ^]^ and anisotropies of the induced current density (ACID)^[^ ^64^ ^]^ were calculated (Figure 5a,b). The five‐ and seven‐membered rings of the sesquifulvalene unit display low NICS(1)_zz values of −0.66 and 2.73 ppm, thus indicating non‐aromaticity. For the two five‐membered rings that are fused with the seven‐membered ring, positive shifts of 12.63 and 14.68 ppm refer to weak anti‐aromaticity. In contrast, for all benzenoid rings, **2** negative NICS(1)_zz values were calculated, denoting aromaticity. The ACID plot of **2** is consistent with the NICS calculations. The clockwise diatropic ring current is mainly delocalized in the peripheral and central regions of the molecule, excluding the five‐ and seven‐membered rings.

Furthermore, the charge transfer between **2** and Au(111) as well as the electrostatic potential surface of free‐standing **2** were analyzed (Figures 5c and S11). Differential charge density analysis (see Figure 5c for top and side view) revealed that the charge is mainly transferred from the periphery of **2** to the Au(111) surface. The electrostatic potential surface exhibits a negative electrostatic potential at the upper fluorene unit (6–5–6) of **2**, denoted in red (Figure S11). This can be explained by the dipole moment of the sesquifulvalene unit with a negative polarization at the five‐membered ring according to Hückel aromaticity^[^ ^65^ ^]^ and is also reported at the example of acepleiadiene^[^ ^66^ ^]^ as well as non‐benzenoid PAHs containing four‐to eight‐membered rings, including a cyclopentaazulene unit.^[^ ^67^ ^]^

### 2.6 Bulk Pyrolysis

**Scheme 3:**
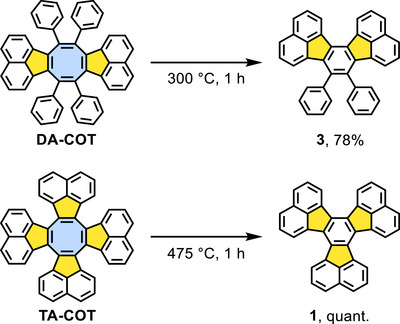
Optimized bulk pyrolysis conditions of **DA‐COT** toward ring‐contracted **3** (top) and **TA‐COT** toward ring‐contracted **1** (bottom) under reduced pressure.

The reactivity of the two COT derivatives on Au(111) was compared to uncatalyzed bulk pyrolysis. **DA‐COT** and **TA‐COT** were pyrolyzed in sealed glass ampoules under reduced pressure, respectively (Scheme 3). The required reaction temperatures were determined by thermogravimetry (TGA)/differential scanning calorimetry (DSC) as 300 °C and 475 °C, respectively (see SI, Figure S7), either via the onset of mass loss (**DA‐COT**) or the pronounced exothermic peak in the DSC (**TA‐COT**). Pyrolysis of **DA‐COT** gave ring contraction product **3** in 78% yield (higher temperatures resulted in decomposition and lower yields; see SI, Table S2), and **TA‐COT** was quantitatively transformed into **1**. Formation of **1** from **TA‐COT** was also observed under Scholl conditions by Yamada et al. in 28% yield.^[^ ^39^ ^]^ In contrast to Au(111)‐catalyzed ring contraction, bulk pyrolysis of **DA‐COT** was not accompanied by cyclodehydrogenation. The reactivity of **TA‐COT** under bulk pyrolysis conditions also differed from the reactivity when adsorbed on Au(111). Only ring contraction upon formation of a six‐membered ring, but no rearrangement toward a sesquifulvalene derivative was observed. Furthermore, the increased reaction temperature by an additional 175 °C of the pyrolytic ring contraction of **TA‐COT** compared to **DA‐COT** parallels the increased strain of the bicyclo[4.2.0]octatriene intermediate and alkyne product. This outcome supports the proposed mechanism of COT electrocyclic reaction with subsequent alkyne extrusion. Nevertheless, in contrast to the Au(111)‐catalyzed reaction, no elimination products could be detected from mass‐spectrometric analysis of the crude pyrolysis products. The literature‐known ring contraction of tetraphenylene to triphenylene at 580 °C^[^ ^68^ ^]^ is also consistent with these results since two Clar sextets are broken up during formation of the corresponding bicyclo[4.2.0]octatriene intermediate, accounting for the increased ring contraction temperature of tetraphenylene.

## 3 Conclusion

We have investigated the reactivity of two COT derivatives, **DA‐COT** and **TA‐COT**, on Au(111) surfaces upon cyclodehydrogenation and compared this process to bulk pyrolysis. The corresponding and desired [8]circulene derivatives were not generated. Instead, a strain‐induced ring contraction of the central COT units toward six‐membered rings was observed on Au(111) for both derivatives **DA‐COT** and **TA‐COT**. Formation of analogous products has already been described during oxidative cyclodehydrogenation of **TA‐COT** under Scholl conditions^[^ ^39^ ^]^ as well as in bulk pyrolysis of tetraphenylene.^[^ ^68^ ^]^ In addition to literature, an electrocyclic reaction of COT toward bicyclo[4.2.0]octatriene with subsequent alkyne extrusion was supposed and verified by detection of an acenaphthylenyl dimer via high‐resolution STM and nc‐AFM. Due to the increased strain of the corresponding bicyclo[4.2.0]octatriene intermediate and bisdehydroacenaphthylene alkyne product, a new reaction pathway opened up for the Au(111)‐catalyzed pyrolysis of **TA‐COT**. This included the rearrangement of **TA‐COT** followed by cyclodehydrogenation toward a non‐benzenoid PAH containing a sesquifulvalene unit. Similar to our observation, a thermal rearrangement of cyclooctadi‐*as*‐indacene derivatives upon FVP was reported, and the reaction mechanism was theoretically analyzed.^[^ ^52^ ^]^ Additionally, we compared the metal‐catalyzed reactions on the surface with bulk pyrolysis, and both derivatives, **DA‐COT** and **TA‐COT**, yielded a ring contraction upon alkyne extrusion. The reaction temperatures were in agreement with the proposed mechanism and confirmed the increased ring strain of intermediates and alkyne products.

## 4 Methods

### 4.1 Pyrolysis Reactions

Pyrolysis reactions were conducted in sealed glass ampoules under reduced pressure that were heated in a muffle furnace. See SI for further details.

### 4.2 Single Crystal and Sample Preparation

Au(111), Ag(111), and Ag(100) surfaces were cleaned by second cycles of Ar^+^ sputtering (pressure: 6.0 × 10^−5^ mbar) and further annealing (Au(111): 780 K, Ag(111)/Ag(100): 760 K). **DA‐COT** was then sublimated onto different surfaces at 470 K and **TA‐COT** at 530 K. Molecular coverage was controlled by evaporation time and temperature.

Reactions were performed by heating the precursors on the surface under a vacuum atmosphere of about 2.0 × 10^−10^ mbar.

### 4.3 STM, nc‐AFM and STS Measurement

All STM images and atomically constant‐height nc‐AFM topologies were captured by CO‐functionalized tips under an ultra‐high vacuum environment of about 2× 10^−11^ mbar and a low temperature of about 4.6 K, which were reached by means of commercial Omicron equipment. CO‐terminated tips were prepared on Cu(111) by utilizing Bartle's method.^[^ ^69^, ^70^ ^]^ nc‐AFM measurements were performed by a qPlus sensor in the constant amplitude mode. The resonant frequency was about 28 KHz, the oscillation amplitude about 70 pm, and the Q factor 13 000∼24 000. STS spectroscopies (d*I*/ d*V* curves) of molecules were explored via lock‐in amplifier to investigate their electronic properties. Processing of all images was implemented by SPIP software.

### 4.4 DFT Calculations

The DFT calculations were performed using the Vienna Ab initio Simulation Package (VASP, version 5.4.4) with the projector‐augmented wave (PAW) method to describe core‐valence interactions.^[^ ^71^, ^72^, ^73^, ^74^ ^]^ The Perdew‐Burke‐Ernzerhof (PBE) functional within the generalized gradient approximation (GGA) was used to model exchange‐correlation effects^[^ ^75^ ^]^ while van der Waals interactions were corrected using the DFT‐D3 method with Becke‐Johnson damping.^[^ ^76^ ^]^ A plane wave basis set with a kinetic energy cutoff of 400 eV was employed, and the reciprocal space was sampled using a Γ‐centered Monkhorst‐Pack scheme with a grid of 1 × 1 × 1.^[^ ^77^ ^]^ Structural optimizations for local minima were conducted to achieve residual forces on atoms below 0.05 eV/Å. The Au(111) surface was modeled using an 8 × 8 unit cell with three atomic layers, and a vacuum thickness of 15 Å was applied along the z direction to eliminate interactions between periodic images. During geometry optimizations, the bottom two layers were fixed while the remaining atoms were allowed to relax.

For calculations of the gas‐phase electronic structure, the Gaussian 16 software package (version A. 03) was employed.^[^ ^78^ ^]^ Geometries of the ground‐state molecules were optimized using the B3LYP hybrid functional with the 6–31G(d) basis set. The electronic structure of the ground‐state molecules was further refined at the B3LYP/6–311G** level. Anisotropy of the induced current density (ACID) plots were generated at the B3LYP/6–311G** level, following Herges’ method.^[^ ^64^ ^]^ NICS values were calculated at the B3LYP/6–311G** level, employing the gauge‐independent atomic orbital (GIAO) approach.^[^ ^62^ ^]^

STM image simulations were performed using the Hive program.^[^ ^79^ ^]^ AFM image simulations were conducted with the “Probe Particle Model” software^[^ ^80^, ^81^ ^]^ where the CO tip was assigned a lateral stiffness of 0.5 N·m⁻¹ and an oscillation amplitude of 2 Å.

## Supporting Information

Supporting Information includes the organic synthesis of the precursors **DA‐COT** and **TA‐COT**, along with their analytical data, such as NMR spectra and crystallographic data. Reaction mechanisms and DFT calculations on strain energy and electrostatic potential energy distribution as well as reactions on Ag(111) and Ag(100) are also presented. The authors have cited additional references within the Supporting Information.^[^ ^82^, ^83^, ^84^ ^]^

## Conflict of Interest

The authors declare no conflict of interest.

## Supporting information



Supporting Information

## Data Availability

The data that support the findings of this study are available from the corresponding author upon reasonable request. Deposition Number CCDC 2399881 (for **DA‐COT**) contains the supplementary crystallographic data for this paper. These data are provided free of charge by the joint Cambridge Crystallographic Data Centre and Fachinformationszentrum Karlsruhe Access Structures service.
