# Supplementary material for: Ring Contraction of Cyclooctatetraenes toward Non‐Benzenoid Polycyclic Aromatic Hydrocarbons by Au(111)‐Catalysis and Bulk Pyrolysis
Source: Chemistry. 2025 Jun 27;31(40):e202501101. doi: 10.1002/chem.202501101 (PMC12271993; doi:10.1002/chem.202501101)
Supplement: Supplementary file 1 — Supporting Information [file CHEM-31-e202501101-s001.docx]

**Supporting Information**

**Table of Contents**

[1 Experimental Procedures 2](#_Toc199806163)

[1.1 General Remarks 2](#_Toc199806164)

[1.2 Synthesis and Characterization 4](#_Toc199806165)

[1.2.1 Procedures 4](#_Toc199806166)

[1.2.2 NMR Spectra 8](#_Toc199806167)

[1.2.3 Crystallographic Data 10](#_Toc199806168)

[2 Results and Discussion 11](#_Toc199806169)

[2.1 Surface Experiments 11](#_Toc199806170)

[2.2 Proposed Mechanisms for the Ring Contraction and Rearrangement Reactions 12](#_Toc199806171)

[2.3 Bulk Thermolysis 13](#_Toc199806172)

[2.4 Computational Studies 14](#_Toc199806173)

[2.4.1 Strain Energies 14](#_Toc199806174)

[2.4.2 STM and AFM Simulations 17](#_Toc199806175)

[2.4.3 Electrostatic Potential Energy Distribution 18](#_Toc199806176)

[3 References 18](#_Toc199806177)

# 1 Experimental Procedures

## 1.1 General Remarks

**Reagents and Solvents for Synthesis**

All reagents and solvents were obtained from commercial suppliers (SIGMA-ALDRICH, Thermo Fisher Scientific, Alfa Aesar, BLDpharm, MERCK, Honeywell, TCI, ACROS, Grüssing and ABCR GmbH) and used without further purification. Deuterated solvents for NMR analysis were purchased from SIGMA-ALDRICH GmbH. Anhydrous solvents were obtained from the solvent purification system MBRAUN MB SPS 800 or stored under an argon atmosphere over molecular sieve (pore diameter 3 Å). All reactions requiring exclusion of oxygen and water were performed in heat-dried glassware under a dry and oxygen-free argon atmosphere by means of Schlenk techniques.

**Flash Column Chromatography**

Flash Column Chromatography was performed using silica gel from SIGMA-ALDRICH GmbH (particle size: 0.04-0.063 mm).

**Thin Layer Chromatography**

Thin layer chromatography (TLC) was performed on TLC plates (POLYGRAM^®^ XTRA SIL G/UV_254_ (silica gel, layer thickness 0.2 mm)) by Macherey-Nagel. Samples were visualized under UV light (254 and 365 nm).

**Infrared Spectroscopy**

IR spectra were recorded from neat compounds on a JASCO FT/IR-4100 spectrometer at room temperature. The data were processed using JASCO Spectra Manager TM II and signals are reported in wavenumbers [cm^-1^].

**Mass Spectrometry**

High-resolution mass spectra (HRMS) were obtained by matrix-assisted laser desorption ionization (MALDI) using DCTB as matrix or electron ionization (EI) experiments under the direction of
Dr. J. H. Gross (Heidelberg University) on following instruments: Bruker ApexQe FT-ICR spectro­meter or JEOL AccuTOF GCx spectrometer.

**Melting Points**

Melting points were determined in open glass capillaries with a Melting Point Appartus MEL-TEMP, Electrothermal, Rochford, UK.

**Nuclear Magnetic Resonance Spectroscopy (NMR)**

All NMR spectra were recorded in deuterated solvents at room temperature on a BRUKER Avance III 300 (300 MHz), a BRUKER Avance III 400 (400 MHz), BRUKER Avance Neo (700 MHz). The chemical shifts δ are given in parts per million (ppm) and referenced to internal solvent signals.^[S1]^ Coupling constants *J* are absolute values and given in Hertz (Hz). The following abbreviations describe the signal multiplicities: dd = doublet of doublet, m = multiplet. NMR spectra were visualized, processed and interpreted with MestReNova v14.0.1-23559 by Mestrelab Research S.L.

**X-Ray Single-Crystal Structure Analysis**

X-ray single-crystal structure analyses were measured on a BRUKER Smart APEX-II Quazar Area Detector diffractometer under the direction of Dr. F. Rominger (Heidelberg University). Diffraction intensities were corrected for Lorentz and polarization effects. An empirical absorption correction was applied using SADABS based on the Laue symmetry of reciprocal space. Heavy atom diffractions were solved by direct methods and refined against F^2^ with the full matrix least square algorithm. Hydrogen atoms were either isotropically refined or calculated. The structures were solved and refined using the SHELXTL software package. Stick models of structures and packing were visualized using Mercury 4.1.0.

**Bulk Pyrolysis reactions**

Bulk pyrolysis reactions were carried out either in a Mettler Toledo TGA/DSC under nitrogen atmosphere (heating rate 10 K/min) or in glass ampoules sealed under reduced pressure in a preheated muffle furnace by PYROTEC Brennofenbau (Osnabrück, Germany).

**DFT Calculations**

Structures were optimized at the B3LYP/def2SVP level of theory using Gaussian16.^[S2]^

**Schemes and Structures**

All schemes and chemical structures drawn in this article were generated by ChemDraw^®^ Professional 23.0.1.10 (64-bit) by Revvity.

## 1.2 Synthesis and Characterization

### 1.2.1 Procedures

Cycloocta[1,2-*a*:3,4-*a*':5,6-*a*'':7,8-*a*''']tetraacenaphthylene (**TA-COT**) was synthesized according to a literature-known procedure.^[S3]^

7,9-Diphenyl-8*H*-cyclopenta[*a*]acenaphthylen-8-one (**6**)

1,3-Diphenylpropan-2-one (5.75 g, 27.4 mmol, 1.00 eq.) and acenaphthylene-1,2-dione (4.98 g, 27.4 mmol, 1.00 eq.) were dissolved in 70 mL of ethanol under reflux. Then, a solution of potassium hydroxide (1.53 g, 27.4 mmol, 1.00 eq.) in 10 mL of ethanol was added and the reaction mixture was stirred under reflux for 15 min. The reaction mixture was cooled down to 0 °C, the resulting precipitate was collected by filtration, washed with cold ethanol and dried under reduced pressure. **6** was obtained as a deep purple solid (7.70 g, 21.6 mmol, 79%).

**^1^H NMR** (301 MHz, CDCl_3_): *δ* (ppm) = 8.10 – 8.03 (m, 2H), 7.90 – 7.80 (m, 6H), 7.63 – 7.48 (m, 6H), 7.45 – 7.37 (m, 2H).

All analytical data are in accordance with literature.^[S4]^

Acenaphthylene-1,2-diylbis(phenylmethanone) (**7**)

According to a literature procedure^[S5]^ 100 g of a microemulsion was prepared by adding an aqueous solution of sodium molybdate dihydrate (121 mg in 5.0 mL water) slowly to a stirred suspension of sodium dodecyl sulfate (7.70 g), *n-*butanol (15.3 g) and DCM (72.0 g) at room temperature.

**6** (1.00 g, 2.81 mmol, 1.00 eq.) was dissolved in 48 g of this microemulsion. The reaction mixture was stirred at room temperature while a hydrogen peroxide solution (35% in water) was added in portions of 630 µL (over 2 d, 3 portions a day). Brine was added and the aqueous phase was extracted with DCM. The combined organic layers were dried over magnesium sulphate and the solvent was removed under reduced pressure. After flash column chromatography (SiO_2_; DCM) **7** was isolated as a yellow solid (903 mg, 2.51 mmol, 89%).

***R_f_*** (SiO_2_, DCM) = 0.41.

**Mp**: 143 °C.

**^1^H NMR** (400 MHz, CDCl_3_): *δ* (ppm) = 8.08 (dd, *J* = 9.5, 7.6 Hz, 4H), 7.72 (dd, *J* = 8.2, 7.0 Hz, 2H), 7.58 – 7.51 (m, 4H), 7.46 – 7.39 (m, 2H), 7.25 – 7.20 (m, 4H).

**^13^C{^1^H} NMR** (101 MHz, CDCl_3_): *δ* (ppm) = 193.8, 141.3, 139.4, 136.8, 133.0, 130.5, 129.1, 129.0, 128.8, 128.8, 128.4, 127.6.

**IR** (ATR): $\tilde{\nu}$ = 1646, 1595, 1500, 1483, 1445, 1421, 1340, 1311, 1279, 1236, 1227, 1192, 1170, 1052, 1000, 963, 904, 822, 768, 755, 709, 696, 688, 664, 643, 616, 594, 558, 498 cm^-1^.

**HRMS** (EI+): *m/z*: [M]^•+^: calcd. for [C_26_H_16_O_2_]^•+^: 360.1145; found: 360.1148 with correct isotope distribution.

(7*Z*,15*Z*)-7,8,15,16-Tetraphenylcycloocta[1,2-*a*:5,6-*a*']diacenaphthylene (**DA-COT**)

In a heat gun dried Schlenk tube under an argon atmosphere titanium(IV) chloride (3.79 g, 19.9 mmol, 7.20 eq.) was added slowly to a mixture of activated zinc powder (2.65 g, 40.5 mmol, 14.6 eq.) in 40 mL of anhydrous and degassed THF at ‑45 °C. The mixture was then stirred under reflux for 2 h. After cooling down to 0 °C, **7** (2.00 g, 5.55 mmol, 2.00 eq.) was added. The reaction mixture was then stirred under reflux overnight. After cooling down to room temperature, an aqueous potassium carbonate solution was added and the aqueous phase was extracted with DCM. The combined organic layers were dried over magnesium sulphate and the solvent was removed under reduced pressure. After flash column chromatography (SiO_2_; PE/THF 95:5, *v*/*v*) **DA-COT** was isolated as an orange solid (1.22 g, 1.86 mmol, 67%).

***R_f_*** (SiO_2_; PE/THF 95:5, *v*/*v*) = 0.10.

**Mp**: 322 °C (decomposition).

**^1^H NMR** (700 MHz, CDCl_3_): *δ* (ppm) = 7.61 – 7.58 (m, 4H), 7.47 – 7.44 (m, 8H), 7.42 – 7.39 (m, 4H), 7.36 – 7.33 (m, 4H), 7.16 – 7.10 (m, 12H).

**^13^C{^1^H} NMR** (176 MHz, CDCl_3_): *δ* (ppm) = 143.0, 140.9, 140.0, 139.2, 130.7, 129.4, 128.2, 128.1, 127.7, 127.0, 126.9, 123.8.

**IR** (ATR): $\tilde{\nu}$ =3051, 2955, 1597, 1489, 1482, 1429, 1262, 1180, 1070, 1028, 910, 821, 769, 760, 721, 696, 691, 674, 639, 634, 619, 597, 585, 567, 558, 548, 500 cm^-1^.

**HRMS** (MALDI+): *m/z*: [M]^•+^: calcd. for [C_52_H_32_]^•+^: 656.2499; found: 656.2494 with correct isotope distribution.

**Crystal data**

Single crystalline specimen were obtained by slow diffusion of methanol into a chloroform solution of **DA-COT**.

Orange crystal (plate), dimensions 0.325 x 0.082 x 0.030 mm^3^, crystal system monoclinic, space group P2_1_/c, Z = 8, a = 20.8378(8) Å, b = 21.0399(8) Å, c = 18.2684(7) Å, α = 90°, β = 93.1659(11)°, γ = 90°, V = 7997.1(5) Å^3^, ρ = 1.289 g/cm^3^, T = 200(2) K, θ_max_= 24.559°, 74493 reflections measured, 13377 unique (R(int) = 0.0617), 8295 observed (I > 2σ(I)), μ = 0.27 mm^‑1^, T_min_ = 0.92, T_max_ = 0.96, 1031 parameters refined, hydrogen atoms were treated using appropriate riding models, goodness of fit 1.03 for observed reflections, final residual values R1(F) = 0.072, wR(F^2^) = 0.167 for observed reflections, residual electron density ‑1.06 to 0.94 eÅ^‑3^.

4,5-Diphenylacenaphtho[1,2-*j*]fluoranthene (**3**)

**DA-COT** (30.0 mg, 45.7 µmol, 1.00 eq.) was filled into a glass ampoule and back-filled thrice with argon after evacuation. After final evacuation, the sealed ampoule was heated in a muffle furnace at 300 °C for 1 h. After flash column chromatography (SiO_2_, PE → PE/DCM 95:5, *v*/*v*) **3** was isolated as a yellow solid (17.0 mg, 35.5 µmol, 78%).

**^1^H NMR** (301 MHz, CDCl_3_): *δ* (ppm) = 8.82 – 8.76 (m, 2H), 7.95 – 7.88 (m, 2H), 7.84 – 7.74 (m, 4H), 7.36 – 7.27 (m, 12H), 6.50 – 6.44 (m, 2H).

All analytical data are in accordance with literature.^[S6]^

Diacenaphtho[1,2-*j*:1',2'-*l*]fluoranthene (**1**)

**TA-COT** (30.0 mg, 49.9 µmol, 1.00 eq.) was filled into a glass ampoule and back-filled thrice with argon after evacuation. After final evacuation, the sealed ampoule was heated in a muffle furnace at 475 °C for 1 h. The resulting solid was washed with DCM to yield **1** as a deep brown solid (22.5 mg, 49.9 µmol, quant.).

**^1^H NMR** (301 MHz, CDCl_3_): *δ* (ppm) = 8.86 – 8.79 (m, 6H), 8.02 – 7.93 (m, 6H), 7.89 – 7.77 (m, 6H).

All analytical data are in accordance with literature.^[S7]^

### 1.2.2 NMR Spectra


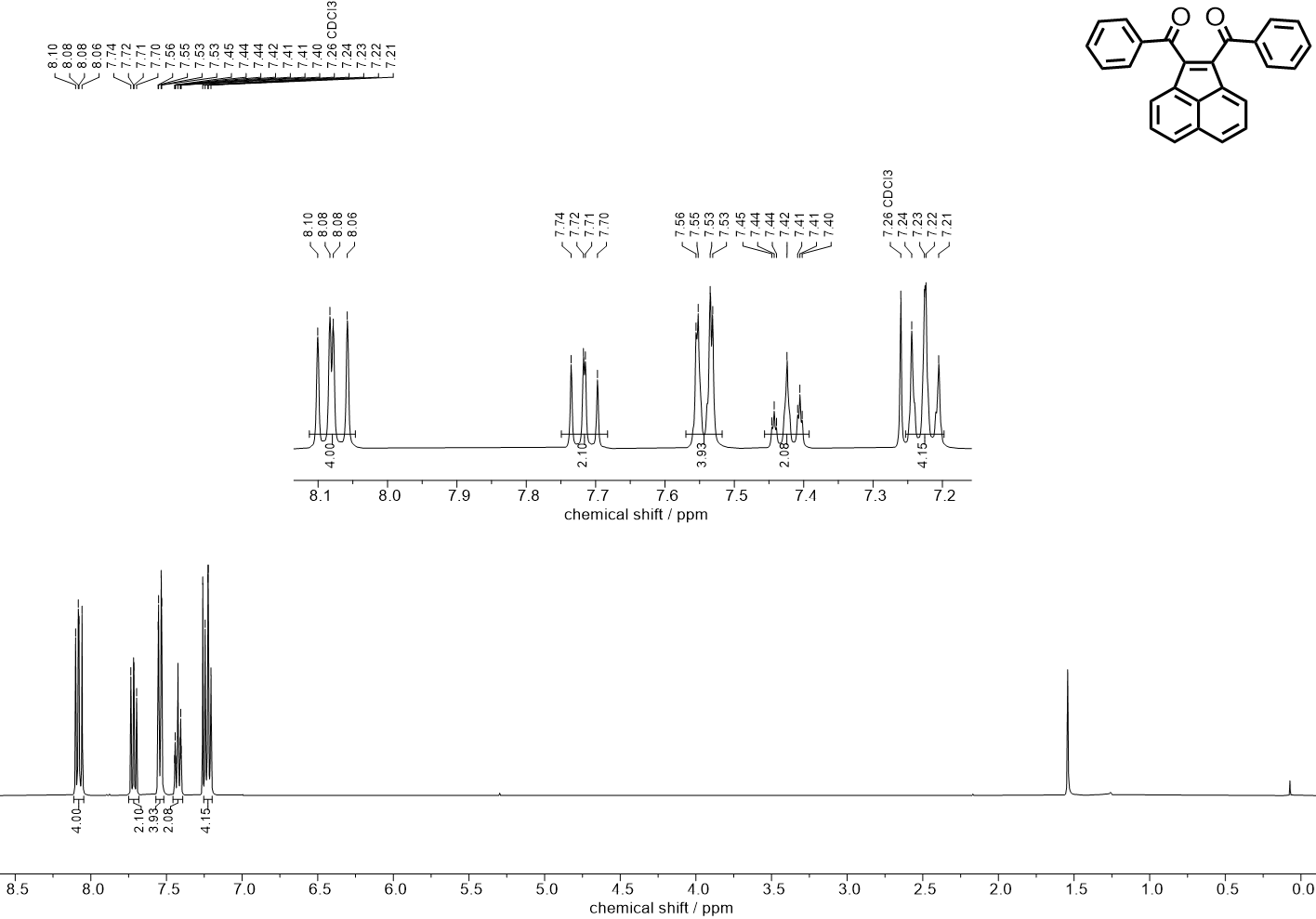


**Figure S1.** ^1^H NMR spectrum (400 MHz) of **7** in CDCl_3_.


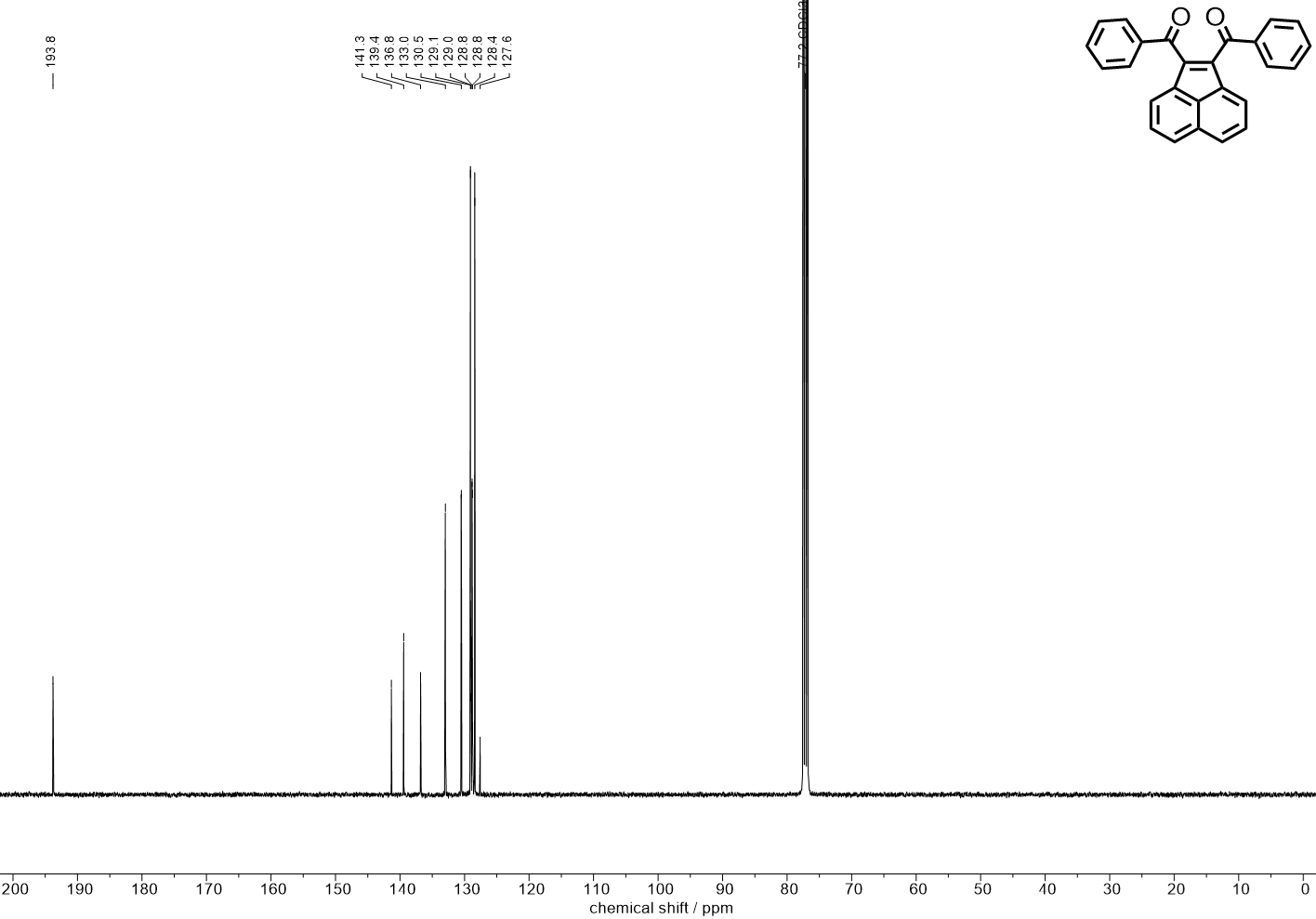


**Figure S2.** ^13^C{^1^H} NMR spectrum (101 MHz) of **7** in CDCl_3_.


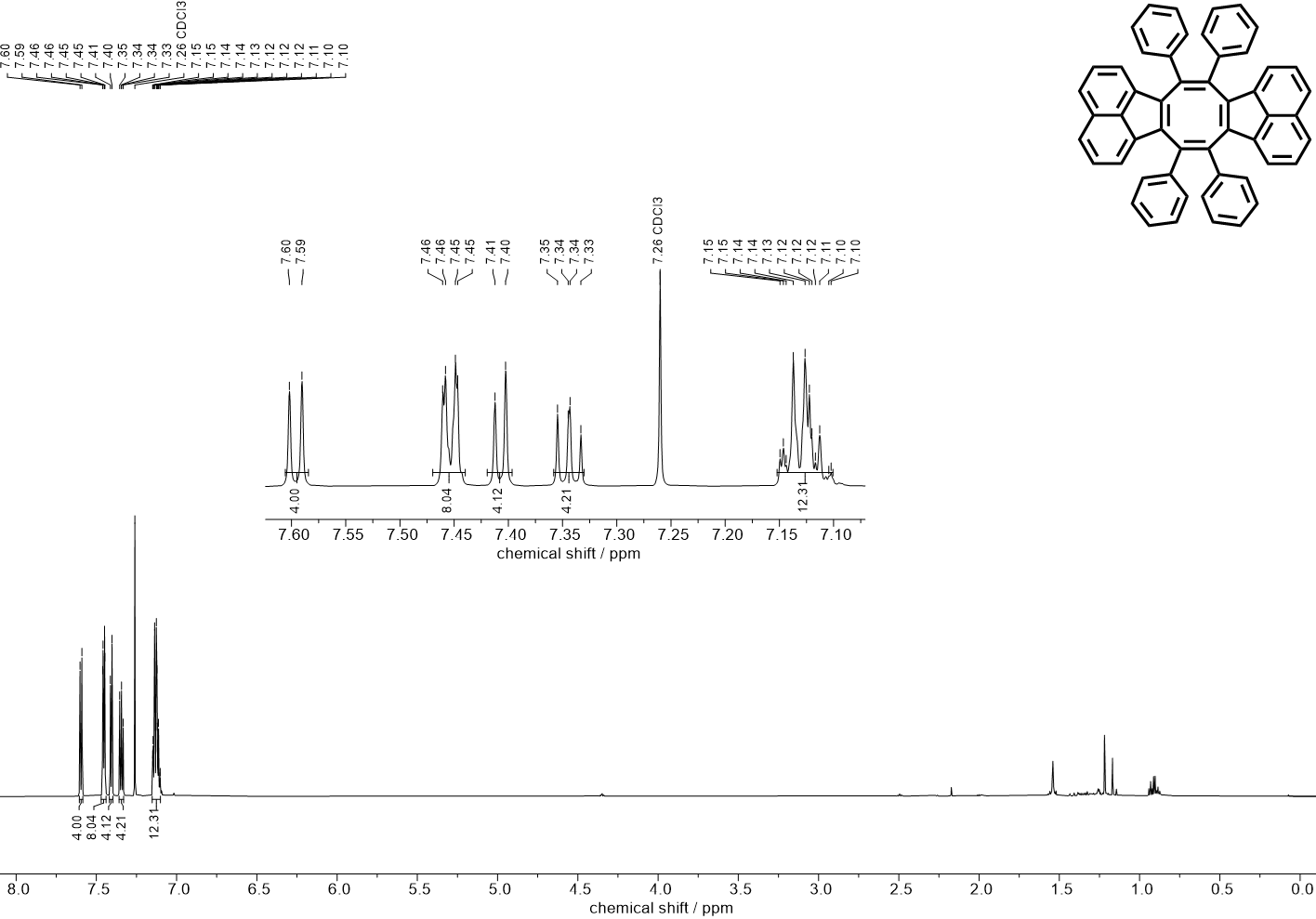


**Figure S3.** ^1^H NMR spectrum (700 MHz) of **DA-COT** in CDCl_3_.


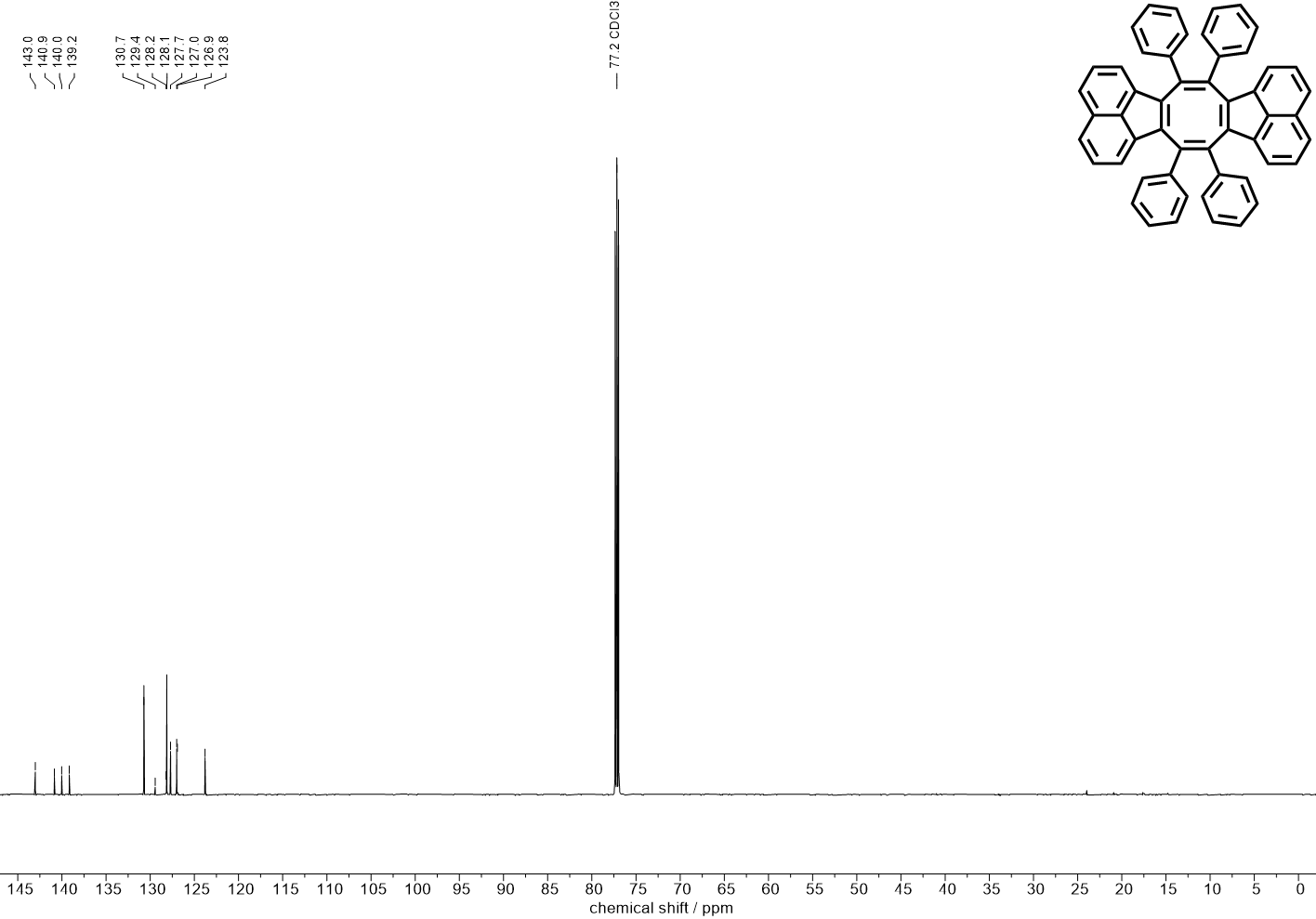


**Figure S4.** ^13^C{^1^H} NMR spectrum (176 MHz) of **DA-COT** in CDCl_3_.

### 1.2.3 Crystallographic Data

**Table S1.** Crystal data and structure refinement for **DA-COT** (CCDC number 2399881).

Empirical formula C_53_H_33_Cl_3_

Formula weight 776.14

Temperature 200(2) K

Wavelength 0.71073 Å

Crystal system monoclinic

Space group P2_1_/c

Z 8

Unit cell dimensions a = 20.8378(8) Å α = 90 deg.

b = 21.0399(8) Å β = 93.1659(11) deg.

c = 18.2684(7) Å γ = 90 deg.

Volume 7997.1(5) Å^3^

Density (calculated) 1.29 g/cm^3^

Absorption coefficient 0.27 mm^-1^

Crystal shape plate

Crystal size 0.325 x 0.082 x 0.030 mm^3^

Crystal colour orange

Theta range for data collection 1.0 to 24.6 deg.

Index ranges -24≤h≤24, -24≤k≤24, -21≤l≤21

Reflections collected 74493

Independent reflections 13377 (R(int) = 0.0617)

Observed reflections 8295 (I > 2σ(I))

Absorption correction Semi-empirical from equivalents

Max. and min. transmission 0.96 and 0.92

Refinement method Full-matrix least-squares on F^2^

Data/restraints/parameters 13377 / 72 / 1031

Goodness-of-fit on F^2^ 1.03

Final R indices (I>2sigma(I)) R1 = 0.072, wR2 = 0.167

Largest diff. peak and hole 0.94 and -1.06 eÅ^-3^

# 2 Results and Discussion

## 2.1 Surface Experiments


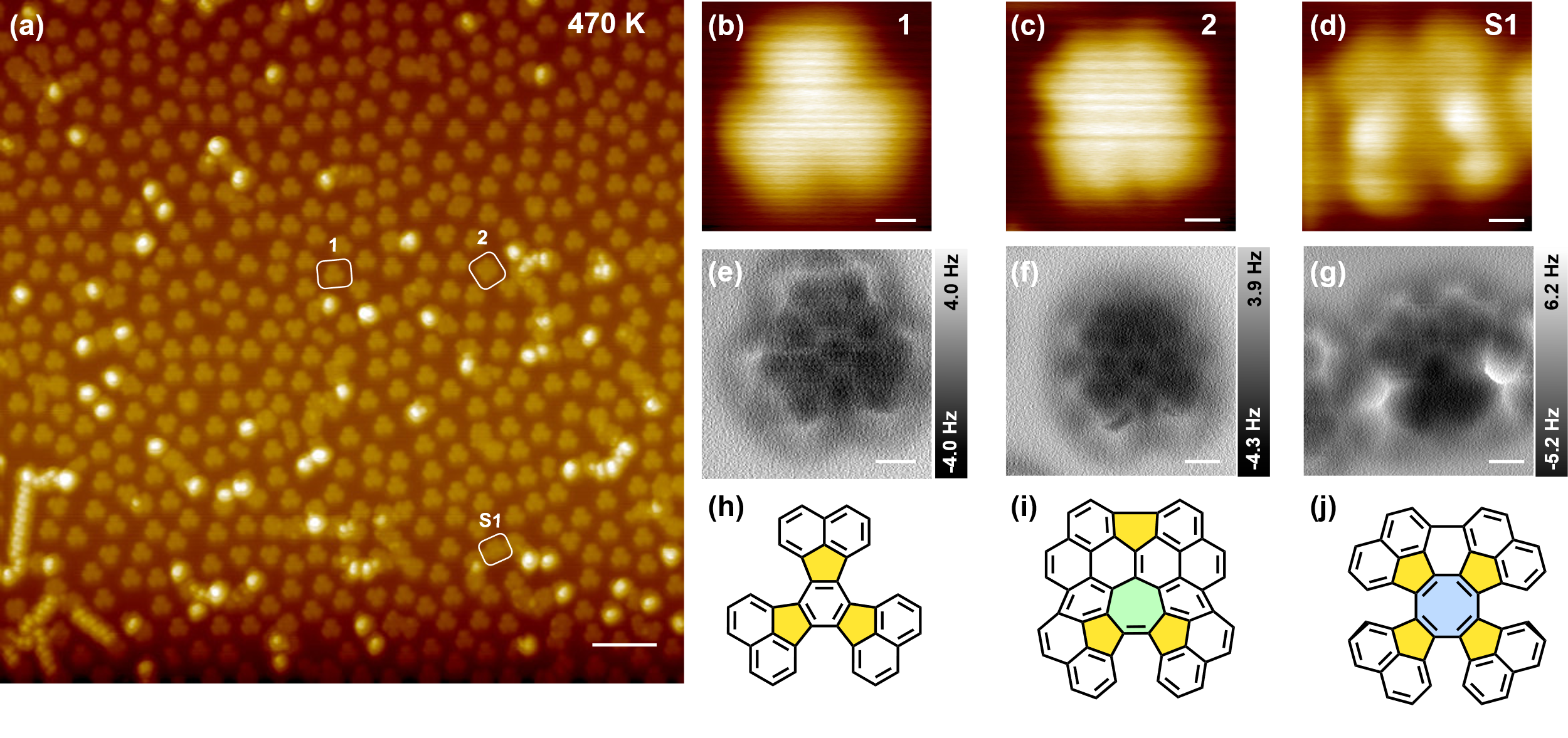


**Figure S5.** Reaction of **TA-COT** after annealing to 470 K on Ag(111). (a) Large-scale STM images of the reaction products. (b-d) High-resolution STM images of individual ring condensation product **1**, ring rearrangement product **2**, and product **S1** after dehydrocyclization between two acenaphthene groups. Products **1**, **2** and **S1** are marked by white rectangles. (e-g) High-resolution AFM images corresponding to reaction products **1**, **2**, and **S1**. (h-j) Chemical structures of above individual products. STM scanning parameters: (a) V*_bias_* = -1 V, I = 10 pA; (b-d) V*_bias_* = -1 V, I = 5 pA. Nc-AFM imaging heights: (e) ∆Z = -10 pm, (f) ∆Z = -80 pm, and (g) ∆Z = -80 pm. Scale bars: (a) 5 nm, (b-g) 0.3 nm.


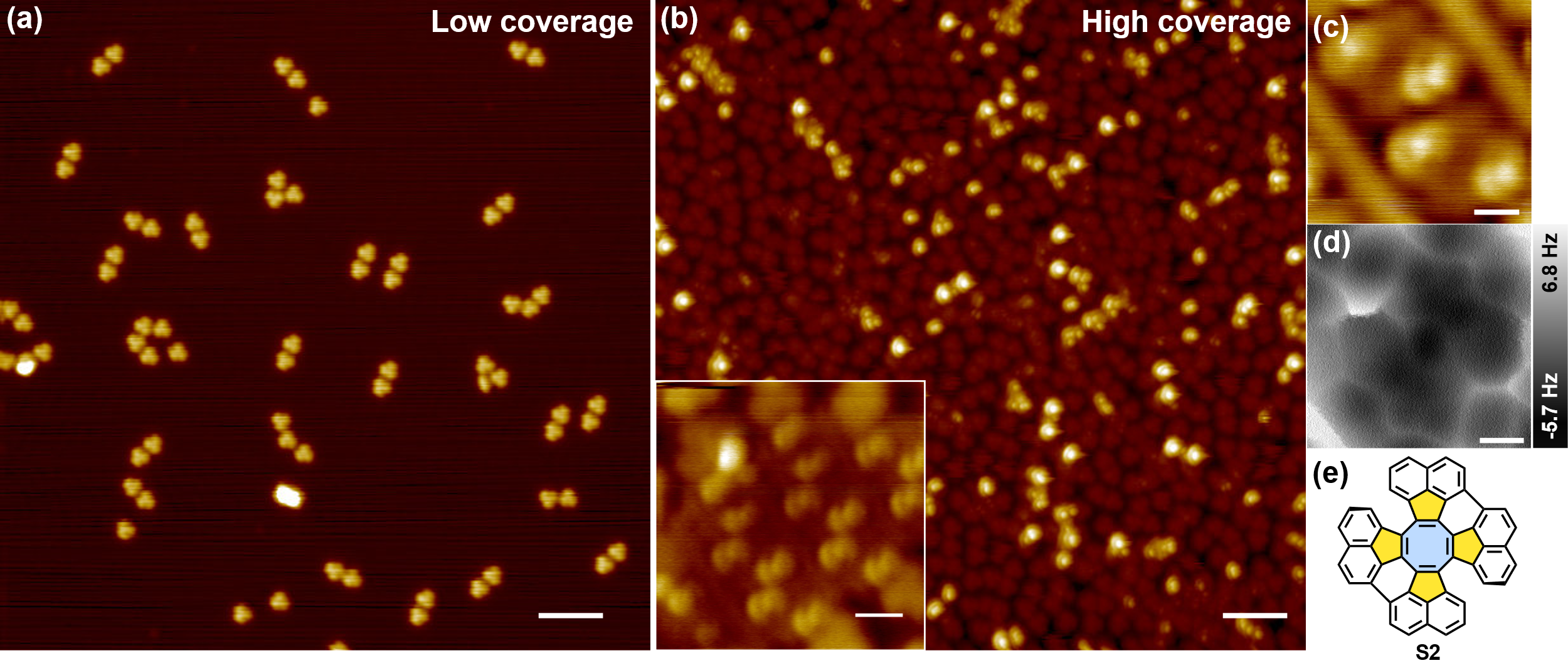


**Figure S6.** Reaction of **TA-COT** on Ag(100) after annealing to 470 K. (a) Large-scale STM image of the product at low coverage. (b) Wide-range STM image of the product at high coverage. The inset: Magnified STM image of one part. (c) STM images of two isolated products. (d, e) Atomically resolved constant-height nc-AFM images of individual molecules in the inset of (b) and the corresponding molecular structures. STM scanning parameters: (a, b) V*_bias_* = -50 mV, I = 8 pA; inset in (b) V*_bias_* = -2 V, I = 2 pA; (c) V*_bias_* = -2 V, I = 2 pA. AFM imaging heights: (d) ∆Z = -170 pm. Scale bars: (a, b) 5 nm, (b) inset 1 nm, (c) 0.5 nm, (d) 0.3 nm.

## 2.2 Proposed Mechanisms for the Ring Contraction and Rearrangement Reactions

**Scheme S1.** Proposed mechanism for the ring contraction of **DA-COT** and **TA-COT** involving valence isomerization and (cyclo)alkyne elimination.

**Scheme S2.** Proposed mechanism of the rearrangement and cyclodehydrogenation of **TA-COT** to sesquifulvalene **2**. **2** and **13** were observed via STM and nc-AFM.

## 2.3 Bulk Thermolysis


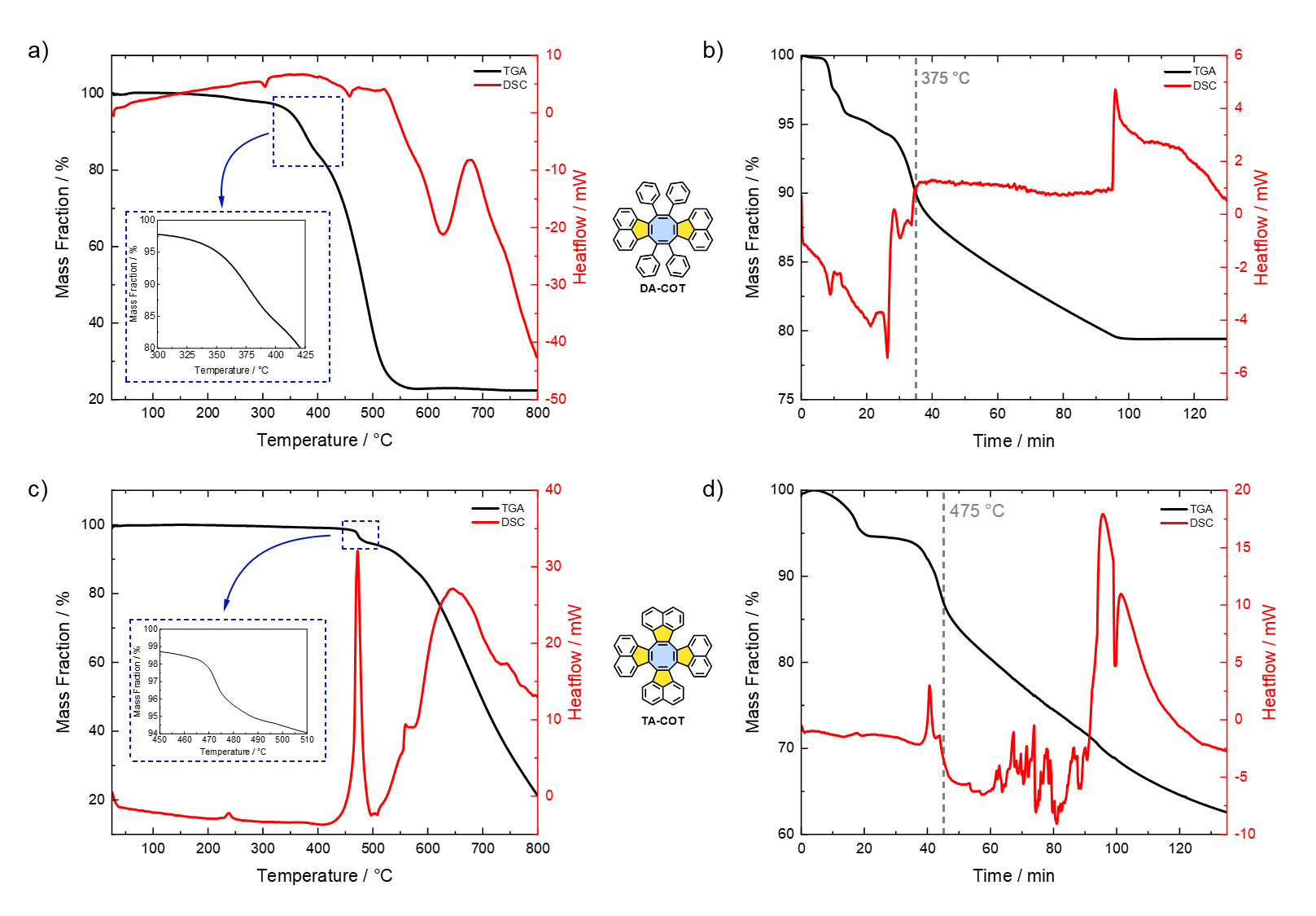


**Figure S7.** Thermogravimetric analysis (TGA) and differential scanning calorimetry (DSC) studies of **DA-COT** (a) in the temperature range 25 – 800 °C (10 K/min), (b) heating up to 375 °C (10 K/min) and holding for 90 min); and **TA-COT** (c) in the temperature range 25 – 800 °C (10 K/min) and d) heating up to 475 °C (10 K/min) and holding for 90 min).

**Table S2.** Tested conditions for bulk thermolysis of **DA-COT**.

|  | | |
| --- | --- | --- |
| **Entry** | **Conditions** | **Results** |
| 1 | 375 °C, 3 h | 23% **3**, decomposition |
| 2 | 375 °C, 1 h | 27% **3**, decomposition |
| 3 | 300 °C, 1 h | 78% **3**, decomposition |
| 4 | 275 °C, 1 h | **3** (traces), reactant (**DA-COT**) |

## 2.4 Computational Studies

### 2.4.1 Strain Energies

Relative strains for **DA-COT** and **TA-COT** were estimated by the reaction depicted in Scheme S3. The structures were optimized at the B3LYP/def2SVP level of theory. Then, a frequency analysis was performed at the B3LYP/def2SVP level of theory. The sum of electronic and zero-point energy was used for the following energy calculations (see Table S3 for energy values). Relative energies of intermediates **10** and **S4** as well as products **3**+**tolane** and **1**+**S2** plotted in Figure S8 are referenced to **DA-COT** (relative energy = 0 kJ•mol^-1^). The relative strain energies *E*_strain_ (equations 1-3, see Table S4 for energy values) were estimated as the reaction energy of the hypohomodesmotic reactions in Scheme S3:

$E_{strain}\left( \boldsymbol{TA-COT} \right)=E\left( \boldsymbol{TA-COT} \right)+2E\left( \boldsymbol{ethene} \right)-E\left( \boldsymbol{DA-COT} \right)$ (equation 1)

$E_{strain}\left( \boldsymbol{S}\boldsymbol{6} \right)=E\left( \boldsymbol{S}\boldsymbol{6} \right)+2E\left( \boldsymbol{ethene} \right)-E\left( \boldsymbol{10} \right)$ (equation 2)

$E_{strain}\left( \boldsymbol{1+S}\boldsymbol{4} \right)=E\left( \boldsymbol{1} \right)+E\left( \boldsymbol{S}\boldsymbol{4} \right)+2E\left( \boldsymbol{ethene} \right)-E\left( \boldsymbol{3} \right)-E\left( \boldsymbol{tolane} \right)$ (equation 3)

**Scheme S3.** Reaction used to estimate the relative strains of **DA-COT** and **TA-COT**.

**Table S3.** Sum of electronic and zero-point energy values calculated at the B3LYP/def2SVP level of theory.

| **Compound** | **Sum of electronic and zero-point energy / Hartree** | **Sum of electronic and zero-point energy / kJ•mol^-1^** |
| --- | --- | --- |
| **ethene** | -78.480889 | -206051.574 |
| **DA-COT** | -1998.697428 | -5247580.097 |
| **10** | -1998.682577 | -5247541.106 |
| **3** | -1459.833296 | -3832792.319 |
| **tolane** | -538.887802 | -1414849.924 |
| **TA-COT** | -1841.725099 | -4835449.247 |
| **S6** | -1841.659717 | -4835277.587 |
| **1** | -1381.329446 | -3626680.460 |
| **S4** | -460.249712 | -1208385.620 |

**Table S4.** Estimated relative strain energy values *E*_strain_ according to equations 1-3.

| **Compound** | **Estimated relative strain energy *E*_strain_ / kJ•mol^-1^** |
| --- | --- |
| **TA-COT** | 28 |
| **S6** | 160 |
| **1+S4** | 473 |

**Figure S8.** Relative energies of the reactants, intermediates and products of the ring contraction reaction of **DA-COT** (green) and **TA-COT** (red) including strain energies *E*_strain_. Relative energies are calculated by the sum of electronic and zero-point energy (see Table S3) and referenced to **DA-COT** (relative energy = 0 kJ•mol^-1^). Plotted relative energy values are given in Table S5.

**Table S5.** Relative energy values plotted in Figure S8.

| **Compound** | **Relative energy / kJ•mol^-1^** |
| --- | --- |
| **DA-COT** | 0 |
| **10** | 39 |
| **3+tolane** | -62 |
| **TA-COT** | 28 |
| **S6** | 199 |
| **1+S4** | 411 |

### 2.4.2 STM and AFM Simulations


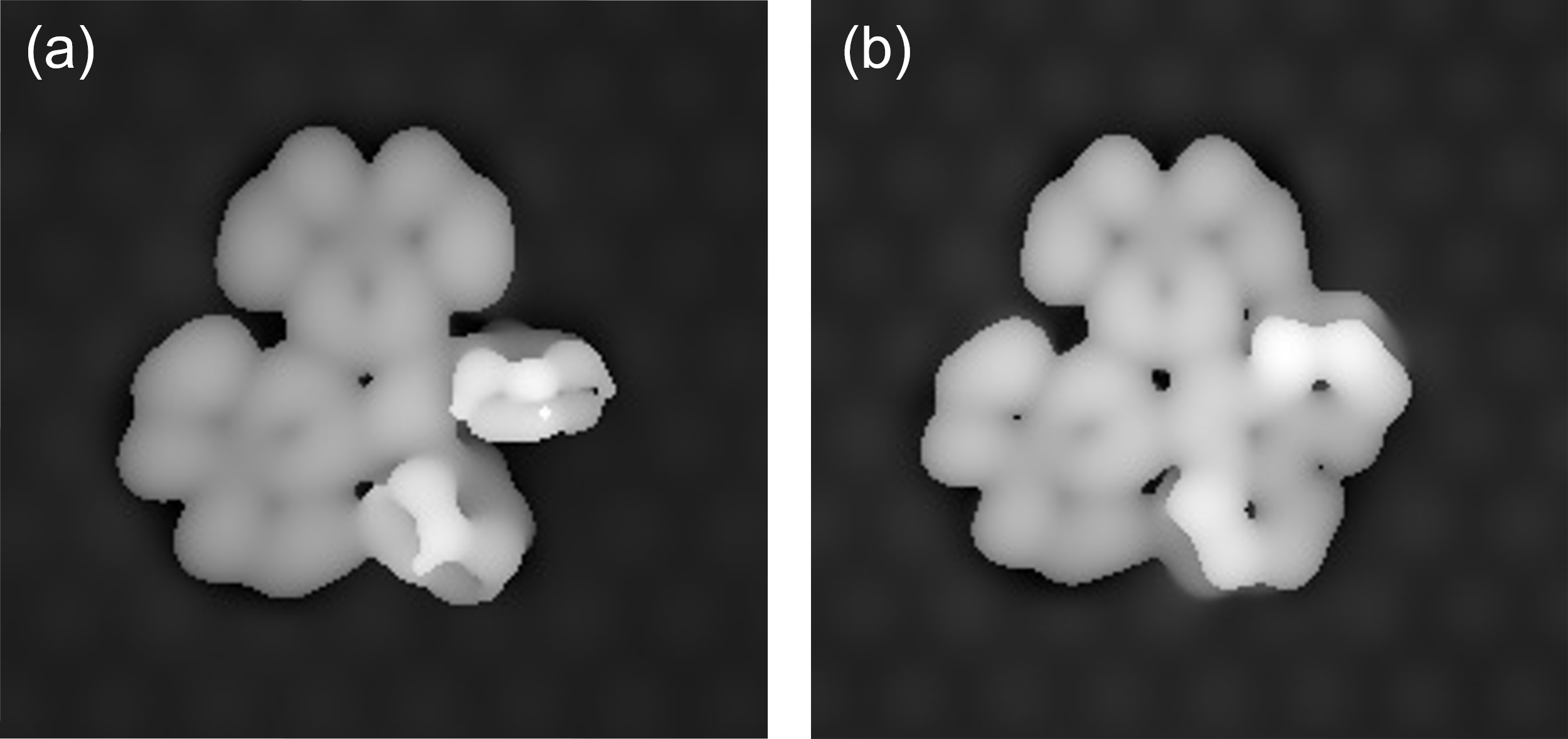


**Figure S9.** STM simulations of intermediates a) **3** and b) **11**.


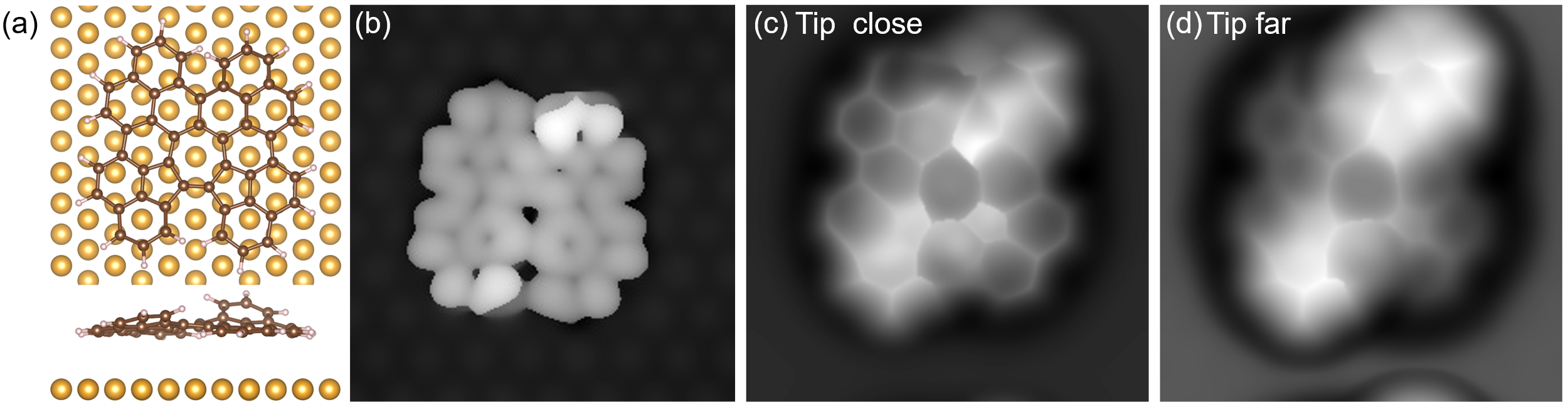


**Figure S10**. a) Top and side views of the optimized structure, b) STM simulation and c, d) AFM simulation with different tip heights of intermediate **13**.

### 2.4.3 Electrostatic Potential Energy Distribution


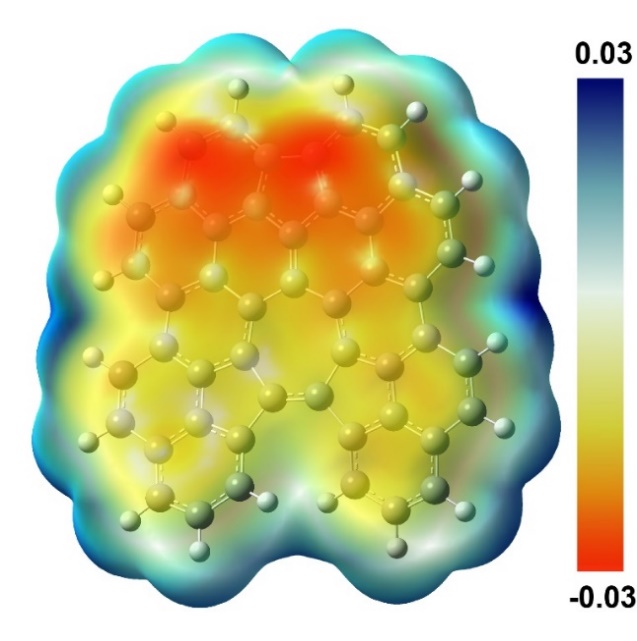


**Figure S11.** Electrostatic potential energy distribution on compound **2** by DFT calculation.

# 3 References

[S1] G. R. Fulmer, A. J. M. Miller, N. H. Sherden, H. E. Gottlieb, A. Nudelman, B. M. Stoltz, J. E. Bercaw, K. I. Goldberg, *Organometallics* **2010**, *29*, 2176-2179.

[S2] Gaussian 16 Rev. C.01, M. J. Frisch, G. W. Trucks, H. B. Schlegel, G. E. Scuseria, M. A. Robb, J. R. Cheeseman, G. Scalmani, V. Barone, G. A. Petersson, H. Nakatsuji, X. Li, M. Caricato, A. V. Marenich, J. Bloino, B. G. Janesko, R. Gomperts, B. Mennucci, H. P. Hratchian, J. V. Ortiz, A. F. Izmaylov, J. L. Sonnenberg, Williams, F. Ding, F. Lipparini, F. Egidi, J. Goings, B. Peng, A. Petrone, T. Henderson, D. Ranasinghe, V. G. Zakrzewski, J. Gao, N. Rega, G. Zheng, W. Liang, M. Hada, M. Ehara, K. Toyota, R. Fukuda, J. Hasegawa, M. Ishida, T. Nakajima, Y. Honda, O. Kitao, H. Nakai, T. Vreven, K. Throssell, J. A. Montgomery Jr., J. E. Peralta, F. Ogliaro, M. J. Bearpark, J. J. Heyd, E. N. Brothers, K. N. Kudin, V. N. Staroverov, T. A. Keith, R. Kobayashi, J. Normand, K. Raghavachari, A. P. Rendell, J. C. Burant, S. S. Iyengar, J. Tomasi, M. Cossi, J. M. Millam, M. Klene, C. Adamo, R. Cammi, J. W. Ochterski, R. L. Martin, K. Morokuma, O. Farkas, J. B. Foresman, D. J. Fox, Gaussian, Inc., Wallingford, CT, **2016**.

[S3] D. P. Sumy, N. J. Dodge, C. M. Harrison, A. D. Finke, A. C. Whalley, *Chem. Eur. J.* **2016**, *22*, 4709-4712.

[S4] K. S. Ivanov, D. E. Samburskiy, L. V. Zargarova, V. Y. Komarov, E. A. Mostovich, *J. Org. Chem.* **2023**, *88*, 11003-11009.

[S5] J.-M. Aubry, S. Bouttemy, *J. Am. Chem. Soc.* **1997**, *119*, 5286-5294.

[S6] R. A. Begum, P. R. Sharp, *Organometallics* **2005**, *24*, 2670-2678.

[S7] A. W. Amick, L. T. Scott, *J. Org. Chem.* **2007**, *72*, 3412-3418.
